# Whole genome wide expression profiles of *Vitis amurensis *grape responding to downy mildew by using Solexa sequencing technology

**DOI:** 10.1186/1471-2229-10-234

**Published:** 2010-10-28

**Authors:** Jiao Wu, Yali Zhang, Huiqin Zhang, Hong Huang, Kevin M Folta, Jiang Lu

**Affiliations:** 1 College of Food Science and Nutritional Engineering, China Agricultural University, Beijing, 100083, China; 2 Horticultural Sciences Department and the Graduate Program in Plant Molecular and Cellular Biology, University of Florida, Gainesville, FL, 32611, USA; 3 School of Information, University of South Florida Tampa, FL, 33620, USA; 4 Center for Viticulture and Small Fruit Research, Florida A&M University, Tallahassee, FL, 32317, USA

## Abstract

**Background:**

Downy mildew (DM), caused by pathogen *Plasmopara viticola *(PV) is the single most damaging disease of grapes (*Vitis *L.) worldwide. However, the mechanisms of the disease development in grapes are poorly understood. A method for estimating gene expression levels using Solexa sequencing of Type I restriction-endonuclease-generated cDNA fragments was used for deep sequencing the transcriptomes resulting from PV infected leaves of *Vitis amurensis *Rupr. cv. Zuoshan-1. Our goal is to identify genes that are involved in resistance to grape DM disease.

**Results:**

Approximately 8.5 million (M) 21-nt cDNA tags were sequenced in the cDNA library derived from PV pathogen-infected leaves, and about 7.5 M were sequenced from the cDNA library constructed from the control leaves. When annotated, a total of 15,249 putative genes were identified from the Solexa sequencing tags for the infection (INF) library and 14,549 for the control (CON) library. Comparative analysis between these two cDNA libraries showed about 0.9% of the unique tags increased by at least five-fold, and about 0.6% of the unique tags decreased more than five-fold in infected leaves, while 98.5% of the unique tags showed less than five-fold difference between the two samples. The expression levels of 12 differentially expressed genes were confirmed by Real-time RT-PCR and the trends observed agreed well with the Solexa expression profiles, although the degree of change was lower in amplitude. After pathway enrichment analysis, a set of significantly enriched pathways were identified for the differentially expressed genes (DEGs), which associated with ribosome structure, photosynthesis, amino acid and sugar metabolism.

**Conclusions:**

This study presented a series of candidate genes and pathways that may contribute to DM resistance in grapes, and illustrated that the Solexa-based tag-sequencing approach was a powerful tool for gene expression comparison between control and treated samples.

## Background

Downy mildew of grapes occurs in most parts of the world where grapes are grown, but favors those regions that experience warm, wet conditions during the vegetative growth of the vine. A major outbreak of the disease can cause severe losses in yield and berry quality. Symptoms of DM are usually first noticed on leaves as yellowish and later oily lesions on the leaf's upper surface with a 'downy' mass observed on the corresponding underside of the leaf. It can also cause deformation of shoots, tendrils, inflorescences and clusters of young berries. Berries become less susceptible as they mature, however rachis infection can spread into the older fruit which leads to direct crop loss by shelling of berries [1].

Downy mildew is caused by the pathogen *Plasmopara viticola *(PV). Primary infection begins with the overwintering oospore on infected leaves or plant litter in the soil that germinates in the spring and produces a sporangium [2]. When plant parts are covered with a film of moisture from rain or irrigation, the sporangium releases small swimming spores (zoospores) that are then spread by splashing water. The spores can germinate by producing a germ tube that enters the green tissue (including leaves, inflorescences, bunches and young berries) through the stomates [3]. Secondary infection, which is the major source of disease spread, produces spores that may be mobilized by wind and rain to establish new infection sites. The cycle ends with the sexual production of over-wintering oospores [2].

Different genotypes of grapes show varying level of resistance to PV, ranging from susceptible *V. vinifera*, to the moderately resistant *V. rupestris *and *V. amurensis*, *V. cinerea*, *V. riparia *and *V. candicans*, to the totally resistant *Muscadinia rotundifolia *[4-6]. The world-wide grape industry relies predominantly on *V. vinifera*, which requires chemical protection to produce healthy fruits. However, such chemicals may have negative environmental impacts and/or pose risk to human health. A promising alternative strategy that could simultaneously improve grape health and limit chemical use is to identify the unique genes or mechanisms from resistant species that could potentially confer resistance to the pathogen or lower presentation of symptoms. These elements may potentially be introduced into *V. vinifera *through long-term breeding efforts or transgenic methods. With this perspective, it is important to unravel the molecular basis of natural defense responses in resistant grapevines to DM challenge, including identification of the genetic processes that may contribute to resistance.

Responses to PV have been characterized in various resistant species. Mechanisms of resistance include induction of chemical barriers, initiation of processes that delay invasive growth of mycelia, or mechanisms that establish hypersensitive response after inoculation of PV [7-9]. Genetic and gene expression profiling studies have concluded that *Rpv1*, NPR1 homologs, and PR protein encoding genes contribute to the function of DM resistance in grapevines [10-12]. Others factors, including the amino acid beta-aminobutyric acid [13], and the proteins beta-1, 3-Glucanase [14], stilbene synthase (STS) [15], phenylalanine ammonia lyase (PAL) [16], thaumatin-like proteins and chitinase [17] may also play an important role in DM resistance. Many attempts, including transgenic [18-21] and traditional breeding approaches [10,22,23], have been undertaken to introgress resistance into *V. vinifera *genotypes.

To understand the mechanism(s) of the host resistance at the molecular level, a critical first step is to identify the transcripts that accumulate in response to the pathogen attack. In this study, "Zuoshan-1", a clonal selection from wild *V. amurensis *with cold hardiness and high resistance to DM [24], was employed to identify a set of candidate genes associated with DM resistance using Solexa sequencing technology. Solexa sequencing is a technology capable of obtaining novel information for whole-genome-wide transcript expression without prior sequence knowledge. This report presents the finding of these tests.

## Results

### Inoculation and symptom development

**Figure 1:**
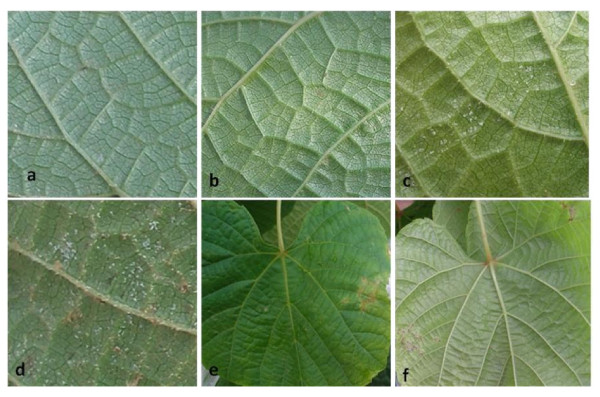
**Symptom development on leaf surface of "Zuoshan-1" after PV infection**. The fourth unfolded leaf from the shoot apex of "Zuoshan-1" was inoculated on (a) day 0. Subsequent images depict the state of infection and symptom development on (b) day 4, (c) day 6, (d) day 8 and (e and f) 18 d. Panel e shows the upper leaf and panel f shows the lower leaf surface.

The fourth unfolded leaf from the shoot apex of "Zuoshan-1" was inoculated with PV. No visible symptoms were observed in the first 4 days (Figure 1a and 1b). The 'downy' mass was obviously observed on the 6th day (Figure 1c) and exacerbated on the 8th day (Figure 1d). Oil spots emerged gradually on the site of pathogen and the spores did not spread to the other healthy tissues 18 days after inoculation (Figure 1e and 1f).

### Tag identification and quantification

A total of 8,549,948 and 7,527,499 tags were sequenced in infected (INF) and control (CON) libraries, respectively (Table 1). After filtering out low quality tags (tags containing 'N' and adaptor sequences), 8,474,583 and 7,525,307 tags (noted herein as "clean" tags) remained in INF and CON libraries. To increase the robustness of the approach, single-copy tags in the two libraries (247,900 in INF and 253,156 in CON library) were excluded from further analysis. As a result, a total of 8,226,683 and 7,272,151 clean tags remained from the two libraries, from which 233,653 (INF) and 203,514 (CON) unique tags were obtained. There were 30,139 more unique tags in the INF than in the CON library, possibly representing genes related to pathogen interaction and symptom development. The percentage of unique tags rapidly declined as copy number increased, indicating only a small portion of the transcripts were expressed at high level in the conditions tested.

**Table 1:** Solexa tags in the infected (INF) and control (CON) libraries.

	INF	CON
total tag	8549948	7527499
clean tag	8474583	7525307
clean tag copy number = 1	247900	253156
unique tag	233653	203514
unique tag copy number >5	98318	80345
unique tag copy number >10	63202	51438
unique tag copy number >20	39772	31441
unique tag copy number >50	19776	14804
unique tag copy number >100	10615	7701

### Depth of sampling

**Figure 2:**
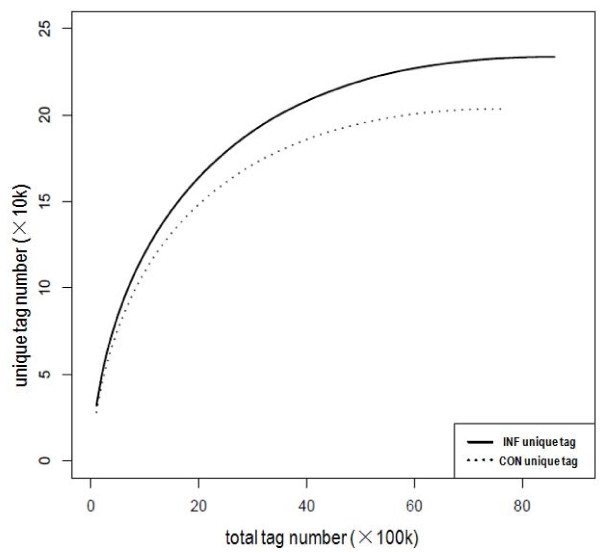
**Accumulation of Solexa total tag and unique tag in the two libraries**. New unique tag ("y" axis) of INF (solid line) and CON (broken line) libraries decreased as the solexa sequencing increased ("x" axis). The total unique tag was 233,653 in INF and 203,514 in CON library.

Saturation of the library is determined by identification of unique tags. Sequencing reaches saturation when no new unique tags are detected. The results shown in Figure 2 indicate that INF and CON libraries were sequenced to saturation, producing a full representation of the transcripts in the conditions tested. In both libraries fewer unique tags were identified as the number of sequencing tags increases, reaching a plateau shortly after 6 M tags were sequenced. No new unique tags were identified as the total tag number approached 8.5 M in INF library and 7.5 M in CON library.

### Annotation analysis of the unique tag

The unique tags were compared against the genome and gene sequences of *V. vinifera *cv. Pinot Noir [25] using blastn. Tags with a complete match or one base pair mismatch were considered further. The results in Table 2 show that a substantial proportion of tags (81.60% in INF library and 83.72% in CON library) matched to the "Pinot Noir" genome, and 91,638 (39.21% of unique tags) and 83,079 (40.82% of unique tags) in INF and CON library matched to 18,841 (61.91%) and 18,068 (59.37%) "Pinot Noir" genes. Further analysis revealed that 82,886 unique tags (35.47%) in INF library and 75,290 (36.99%) in CON library matched to only one gene sequence in the "Pinot Noir' genome (Table 2). These data indicated that approximately 50% of transcripts predicted in grape are expressed in the infected or control leaves, with more transcripts present in the infected sample.

**Table 2:** Annotation of "Zuoshan-1" Solexa tags against the "Pinot Noir" genomic sequence.

	INF		CON	
	
	match to genome	match to gene	match to genome	match to gene
unique tag	190665 (81.60%)*	91638 (39.21%)*	170380 (83.72%)*	83079 (40.82%)*
matched genes		18841 (61.91%)^#^		18068 (59.37%)^#^

unique tag matched to one gene		82886 (35.47%)*		75290 (36.99%)*
matched genes		15249 (50.51%)^#^		14549 (47.81%)^#^

Note: *percentage of matched tags/total tags;^#^percentage of matched genes/total assembled CDs of "Pinot Noir".

Tags with no homology to grape were compared with blastn to the VBI Microbial Database [26] containing genomic sequence information from *Phytophthora sojae*, *Phytophthora infestans *and *Hyaloperonospora parasitica*. There were 251 tags identified in INF library found to be identical to those of the oomycete during PV infection (additional file 1).

### Comparison of gene expression level between the two libraries

**Figure 3:**
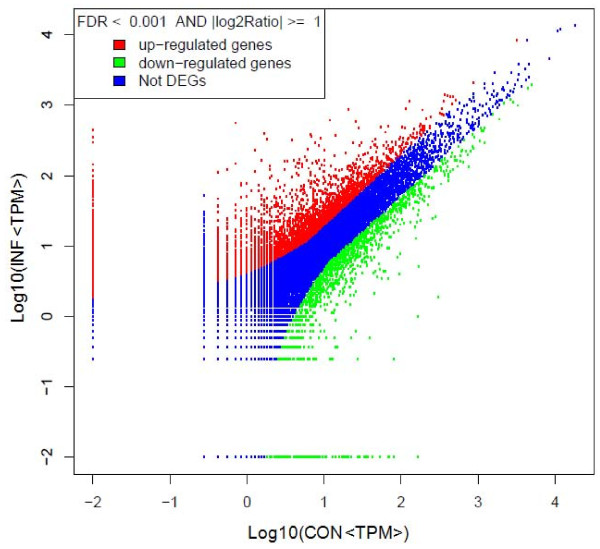
**Comparision of gene expression level between the two libraries**. For comparing gene expression level between the two libraries, each library was normalized to 1 million tags. Red dots represent transcripts more prevalent in the infected leaf library, green dots show those present at a lower frequency in the infected tissue and blue dots indicate transcripts that did not change significantly. The parameters "FDR <0.001" and "log2 Ratio ≥ 1" were used as the threshold to judge the significance of gene expression difference.

**Figure 4:**
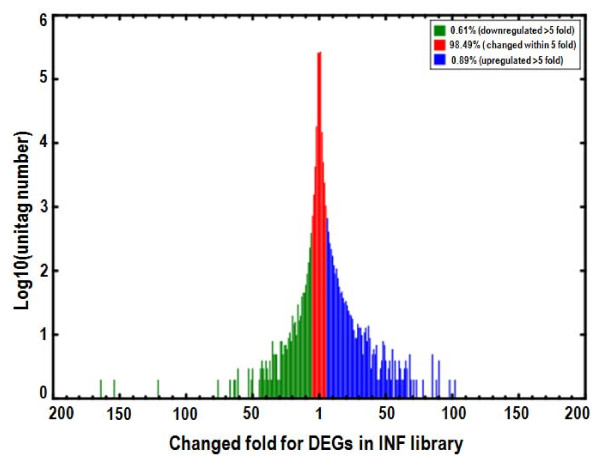
**Differentially expressed tags in infected (INF) tissue library**. The "x" axis represents fold-change of differentially expressed unique tags in the INF library. The "y" axis represents the number of unique tags (log10). Differentially accumulating unique tags with a 5-fold difference between libraries are shown in the red region (98.49%). The blue (0.89%) and green (0.61%) regions represent unique tags that are up- and downregulated for more than 5 fold in the INF library, respectively.

Differences of tag frequencies that appeared in the INF and CON libraries were used for estimating gene expression levels in response to PV infection. The transcripts detected with at least two-fold differences in the two libraries are shown in Figure 3 (FDR <0.001). The red dots (3,125) and green dots (1,847) represent transcripts higher or lower in abundance for more than two fold in INF library, respectively. The blue dots represent transcripts that differed less than two fold between the two libraries, which were arbitrarily designated as "no difference in expression". The DEGs with five fold or greater differences in accumulation were shown in Figure 4. A total of 513 genes (about 0.9% total unique tags) increased by at least five fold, and 167 genes (about 0.6% total unique tags) were decreased by at least five fold in the INF library, while the expression level of 98.5% unique tags was within five-fold difference between the two samples.

Of DEGs with differences greater than twenty fold (Table 3), 69 genes were present at higher levels in the INF library, 67 of which were associated with defense (6), transport (3), transcription (11), signal transduction (14) and metabolism (33). The highest DEG was phosphate-induced protein gene which was present at 229 fold of control levels. Among these highly expressed genes, many were associated with senescence, abiotic and biotic stresses.

**Table 3:** List of DEGs changed for 20 fold and more in INF library.

Gene	Annotation	Stress related function	Accession	Identity	Fold
**Upregulated genes**					
***Defence***					
GSVIVT00025506001	polygalacturonase-inhibiting protein [*Vitis labrusca *x *Vitis Riparia*]	inhibits fungal endopolygalacturonases	ACS16072.1	333/333 (100%)	60
GSVIVT00001105001	thaumatin-like protein [*Vitis vinifera*]	pathogen defence; drought and heat combination	AAQ10092.1	217/225 (96%)	57
GSVIVT00017370001	harpin-induced protein-related/HIN1-related/harpin-responsive protein-related [*Arabidopsis thaliana*]	pathogen defence; senescence	NP_565634.1	141/267 (52%)	33
GSVIVT00002965001	TMV response-related protein [*Zea mays*]	Tobacco Mosaic Virus response	ACG48457.1	39/91 (42%)	32
GSVIVT00005362001	glutaredoxin [*Populus trichocarpa*]	senescence	EEE75685.1	91/155 (58%)	29
GSVIVT00024683001	beta-glucosidase [*Rosa *hybrid cultivar]	activation of phytoanticipins	BAG13451.1	382/531 (71%)	21
***Transport***					
GSVIVT00001094001	multidrug resistance pump, putative [*Ricinus communis*]	fungal resistance	EEF51093.1	407/509 (79%)	121
GSVIVT00015121001	mitochondrial dicarboxylate carrier protein, putative [*Ricinus Communis*]	aluminum tolerance	EEF48606.1	271/324 (83%)	38
GSVIVT00030447001	multidrug resistance protein ABC transporter family protein [*Populus Trichocarpa*]	Senescence; drought and heat combination	EEE80779.1	64/194 (32%)	25
***Signal transduction***					
GSVIVT00030628001	leucine-rich repeat receptor-like protein kinase [*Nicotiana tabacum*]	senescence	AAF66615.1	644/923 (69%)	145
GSVIVT00006178001	FERONIA receptor-like kinase [*Arabidopsis thaliana*]	defence, stresses	ABT18100.1	317/621 (51%)	56
GSVIVT00019504001	MAP3K-like protein kinase [*Arabidopsis thaliana*]	disease resistance, drought and heat combination	CAB16796.1	184/359 (51%)	52
GSVIVT00002706001	calmodulin-binding protein [*Arabidopsis thaliana*]	senescence	NP_565379.1	21/45 (46%)	39
GSVIVT00020989001	calcium-binding EF hand family protein [*Arabidopsis thaliana*]	defence related; senescence; drought and heat combination	NP_568568.1	81/166 (48%)	35
GSVIVT00029809001	ethylene-regulated transcript 2 (ERT2) [*Arabidopsis thaliana*]	senescence	CAB45883.1	96/204 (47%)	34
GSVIVT00036549001	calmodulin-binding protein [*Arabidopsis thaliana*]	senescence	NP_565379.1	149/366 (40%)	28
GSVIVT00002973001	calmodulin binding protein-like [*Elaeis guineensis*]	senescence	ABP04242.1	89/135 (65%)	27
GSVIVT00025017001	BRASSINOSTEROID INSENSITIVE 1-associated receptor kinase 1 precursor, putative [*Ricinus communis*]	disease, cell death	EEF29110.1	415/639 (64%)	26
GSVIVT00000612001	nodulin-like protein [*Arabidopsis thaliana*]	drought and heat combination	AAC28987.1	397/550 (72%)	23
GSVIVT00033036001	RING-H2 subgroup RHE protein [*Populus tremula *x *Populus alba*]	drought and heat combination	AAW33880.1	168/296 (56%)	22
GSVIVT00009150001	PAR-1a [*Nicotiana tabacum*]	potato virus Y, SAR induce	CAA58733.1	127/178 (71%)	22
GSVIVT00027614001	receptor-protein kinase-like protein [*Arabidopsis thaliana*]	drought and heat combination	BAA98098.1	632/849 (74%)	20
GSVIVT00030574001	leucine-rich repeat receptor-like protein kinase [*Arabidopsis thaliana*]	senescence	ACN59244.1	317/611 (51%)	20
***Transcription***					
GSVIVT00014947001	zinc-finger protein 1 [*Datisca glomerata*]	defence, stresses	AAD26942.1	144/246 (58%)	60
GSVIVT00016398001	dehydration-responsive element binding protein 3 [*Glycine max*]	biotic and abiotic stresses	ABB36646.1	116/187 (62%)	52
GSVIVT00007409001	DRE-binding protein 3b [*Gossypium hirsutum*]	drought and heat combination	ABB45861.1	134/237 (56%)	22
GSVIVT00020131001	basic helix-loop-helix protein [*Nicotiana tabacum*]	senescence	BAF30984.1	105/228 (46%)	33
GSVIVT00001092001	Dehydration-responsive element-binding protein 1F, putative [*Ricinus communis*]	phytohormone, pathogen and environmental stresses	EEF51090.1	143/242 (59%)	30
GSVIVT00007410001	CBF4 transcription factor [*Vitis vinifera*]	cold stress	ABE96792.1	218/218 (100%)	30
GSVIVT00016403001	jasmonate ZIM domain 1 [*Catharanthus roseus*]	wounding; herbivory; salinity	ACM89457.1	131/275 (47%)	27
GSVIVT00028041001	AP2 domain class transcription factor [*Malus *x *domestica*]	senescence; drought and heat combination	ADE41117.1	172/327 (52%)	26
GSVIVT00027444001	GRAS family transcription factor [*Populus trichocarpa*]	chitin response	EEE95719.1	446/586 (76%)	26
GSVIVT00006790001	basic helix-loop-helix (bHLH) family protein [*Arabidopsis thaliana*]	fugal resistance related; senescence	NP_568850.1	152/239 (63%)	21
GSVIVT00002446001	WRKY transcription factor 21 [*Populus tomentosa *x *P. bolleana*]	senescence,stresses	ACV92023.1	196/364 (53%)	21
***Metabolism***					
GSVIVT00015203001	putative phosphate-induced protein [*Nicotiana tabacum*]	unidentified	BAA33810.1	243/317 (76%)	229
GSVIVT00016518001	salt responsive protein 2 [*Solanum lycopersicum*]	drought and heat combination	ACG50004.1	309/464 (66%)	165
GSVIVT00024884001	S-adenosyl-L-methionine:salicylic acid carboxyl methyltransferase [*Chimonanthus praecox*]	biotic and abotic stresses	ABU88887.2	191/377 (50%)	97
GSVIVT00024408001	potein-binding protein, putative [*Ricinus communis*]	unidentified	EEF27653.1	393/605 (64%)	87
GSVIVT00028930001	ubiquitin-protein ligase, putative [*Ricinus communis*]	senescence	EEF42248.1	357/602 (59%)	72
GSVIVT00014730001	cytochrome P450 [*Populus trichocarpa*]	senescence; drought and heat combination	EEE73840.1	261/453 (57%)	70
GSVIVT00000988001	9-cis-epoxycarotenoid dioxygenase 1 [*Vitis vinifera*]	senescence; defence	AAR11193.1	602/610 (98%)	62
GSVIVT00023009001	ATPP2-A2, putative [*Ricinus communis*]	unidentified	EEF38353.1	114/158 (72%)	56
GSVIVT00014704001	putative integral membrane protein [*Cyanothece *sp. CCY0110]	unidentified	EAZ88012.1	53/176 (30%)	51
GSVIVT00018424001	tropinone reductase, putative [*Ricinus communis*]	senescence; drought and heat combination	EEF38138.1	194/264 (73%)	48
GSVIVT00032938001	aspartic proteinase nepenthesin-1 precursor, putative [*Ricinus communis*]	phosphorus deficiency; salt stress	EEF29846.1	306/441 (69%)	39
GSVIVT00024072001	protein phosphatase 2c, putative [*Ricinus communis*]	senescence	EEF41194.1	254/393 (64%)	37
GSVIVT00015200001	putative phosphate-induced protein [*Capsicum chinense*]	unidentified	BAG16530.1	186/289 (64%)	37
GSVIVT00022245001	f-box family protein [*Populus trichocarpa*]	senescence	EEE87327.1	139/345 (40%)	37
GSVIVT00016166001	ATP-dependent DNA helicase [*Brevibacillus brevis*]	DNA repair	BAH41662.1	16/45 (35%)	36
GSVIVT00024387001	nucleic acid binding protein, putative [*Ricinus communis*]	oxidative; ABA; abiotic stresses	EEF29282.1	102/164 (62%)	34
GSVIVT00024235001	protein phosphatase 2C [*Nicotiana tabacum*]	senescence	CAC10358.1	257/429 (59%)	34
GSVIVT00035825001	ubiquitin-protein ligase, putative [*Ricinus communis*]	senescence	EEF40124.1	572/719 (79%)	32
GSVIVT00019233001	TPA: isoflavone reductase-like protein 3 [*Vitis vinifera*]	putative defence	CAI56332.1	301/319 (94%)	31
GSVIVT00014029001	TPA_exp: cellulose synthase-like D1 [*Oryza sativa*]	unidentified	DAA01752.1	999/1171 (85%)	31
GSVIVT00007984001	serine acetyltransferase [*Nicotiana plumbaginifolia*]	oxidative stress	AAR18403.1	179/307 (58%)	30
GSVIVT00036225001	Beta-expansin 1a precursor, putative [*Ricinus communis*]	osmotic stress	EEF28288.1	207/259 (79%)	27
GSVIVT00017518001	spotted leaf protein, putative [*Ricinus communis*]	hypersensitive response; cell death; senescence	EEF38265.1	243/402 (60%)	27
GSVIVT00007452001	wound-induced protein WIN2 precursor, putative [*Ricinus communis*]	antifungal	EEF31100.1	142/197 (72%)	26
GSVIVT00002450001	UDP-glucose:glucosyltransferase [*Lycium barbarum*]	drought and heat combination	BAG80556.1	293/464 (63%)	24
GSVIVT00036349001	glucose-1-phosphate adenylyltransferase, putative [*Ricinus communis*]	drought and heat combination	EEF49428.1	412/531 (77%)	24
GSVIVT00028839001	spotted leaf protein, putative [*Ricinus communis*]	hypersensitive response; cell death; senescence	EEF52025.1	385/674 (57%)	24
GSVIVT00009741001	f-box family protein [*Populus trichocarpa*]	senescence	EEE86166.1	93/182 (51%)	24
GSVIVT00019669001	galactinol synthase [*Solanum lycopersicum*]	oxidative stress; drought; salinity; chilling; heat shock	BAH98060.1	231/316 (73%)	24
GSVIVT00030537001	senescence-associated protein, putative [*Medicago truncatula*]	Senescence; drought and heat combination	ABD32641.1	99/144 (68%)	23
GSVIVT00001432001	protein phosphatase 2c, putative [*Ricinus communis*]	senescence; drought and heat combination	EEF34881.1	319/389 (82%)	23
GSVIVT00033193001	galactinol synthase [*Capsicum annuum*]	oxidative stress; drought; salinity; chilling; heat shock	ABQ44212.1	239/315 (75%)	21
GSVIVT00023109001	ATEXO70H4 (exocyst subunit EXO70 family protein H4); protein binding [*Arabidopsis thaliana*]	unidentified	NP_187563.1	331/585 (56%)	21
***various functions***					
GSVIVT00017533001	PREDICTED: hypothetical protein [*Vitis vinifera*]	unidentified	XP_002279648.1	500/500 (100%)	20
GSVIVT00020834001	CW14 [*Arabidopsis thaliana*]	unidentified	BAA87958.1	300/533 (56%)	23
**Downregulated genes**					
***Defence***					
GSVIVT00016961001	Immunoglobulin/major histocompatibility complex [*Medicago truncatula*]	disease resistance	ABP03850.1	426/672 (63%)	-164
GSVIVT00014282001	pathogenesis-related like protein [*Arabidopsis thaliana*]	defence	AAM66077.1	117/215 (54%)	-67
***Metabolism***					
GSVIVT00027449001	(-)-germacrene D synthase [*Vitis vinifera*]	wounding; methyl jasmonate	AAS66357.1	500/553 (90%)	-164
GSVIVT00027451001	(-)-germacrene D synthase [*Vitis vinifera*]	wounding; methyl jasmonate	AAS66357.1	503/557 (90%)	-150
GSVIVT00027450001	(-)-germacrene D synthase [*Vitis vinifera*]	wounding; methyl jasmonate	AAS66357.1	274/319 (85%)	-53
GSVIVT00027456001	(-)-germacrene D synthase [*Vitis vinifera*]	wounding; methyl jasmonate	AAS66357.1	454/545 (83%)	-22
GSVIVT00014725001	cytochrome P450 [*Populus trichocarpa*]	pathogen induced	EEE73840.1	299/511 (58%)	-41
GSVIVT00014727001	cytochrome P450 [*Populus trichocarpa*]	pathogen induced	EEE73840.1	269/447 (60%)	-35
GSVIVT00007099001	thioredoxin x [*Populus trichocarpa*]	defence; abiotic stresses, senescence	EEE90516.1	98/117 (83%)	-39
GSVIVT00008711001	beta-cyanoalanine synthase [*Betula pendula*]	cyanide metabolism	AAN86822.1	311/352 (88%)	-36
GSVIVT00037489001	non-specific lipid transfer protein [*Vitis vinifera*]	defence related	ABA29446.1	119/119 (100%)	-28
GSVIVT00029445001	expansin [*Vitis labrusca x Vitis vinifera*]	defence related	BAC66695.1	252/252 (100%)	-22
GSVIVT00006300001	UDP-glucosyltransferase, putative [*Ricinus communis*]	defence related	EEF47681.1	268/466 (57%)	-22
***various functions***					
GSVIVT00005678001	male sterility-related protein [*Linum usitatissimum*]	unidentified	ACA28679.1	260/503 (51%)	-23
GSVIVT00032599001	hypothetical protein [*Vitis vinifera*]	unidentified	XP_002284962.1	368/368 (100%)	-22

Fifteen DEGs were less abundant in the INF library. Those present twenty fold or more in the CON library were also listed in Table 3, in which 13 genes were classified as defense (2) and metabolism (11), including genes encoding cytochrome P450 and PR proteins. The greatest differences between INF and CON DEGs were (-)-germacrene D synthase and immunoglobulin/major histocompatibility complex that both were present 164-fold lower in the INF library than in the CON library.

### Real-time RT-PCR analysis

**Figure 5:**
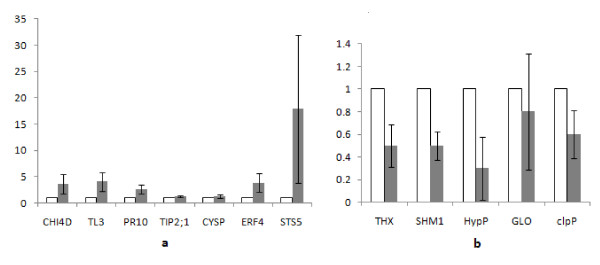
**Real-time RT-PCR analysis for twelve differentially expressed genes**. Real-time RT-PCR analysis for twelve transcripts in control (white) and infected (gray) samples, including (a) seven more abundant in the INF library and (b) five less prevalent in the INF library as identified by Solexa expression profile. All data were normalized to the actin expression level. Data represent fold change of RQ (relative quantification) in infected vs. control samples. Bars represent RQ standard deviation calculated from three biological replicates.

In order to validate Solexa expression profiles, the steady-state transcript levels of 12 "defense related" genes were analyzed. Among them, seven genes (CHI4D, TL3, PR10, TIP2;1, CYSP, ERF4, STS5) were upregulated and five genes (THX, SHM1, HypP, GLO, ClpP) were downregulated (Figure 5). Actin, tested to be stable in our previous work, was chosen as a reference gene for data normalization. The trend of RT-PCR based expression profiles among these selected genes was similar to those detected by Solexa-sequencing based method. However, the scales of difference between the INF and CON were generally smaller in Real-time PCR (1-18 fold differences) than in those detected by the Solexa-sequencing based method (2 - 57 folds) (Table 4).

**Table 4:** Genes selected for Real-time RT-PCR.

Gene	Description	Forward primer	Reverse primer	Target size	Solexa fold	RT-PCR fold
CHI4D	*V. vinifera *class IV chitinase (gb|AF532966.1)	TCCCACGTTCCCCCTTCT	GTAGCTTGGCTGCCATTTTTG	59	11	4
TL3	*V.vinifera *thaumatin-like protein (gb|AF532965.1)	ACCCCACTCCAACCATCAAG	GATTTTGCAGAGGCCCATTG	59	57	4
PR10	Tamnara Tam-RP10 pathogenesis-related protein 10 (dbj|AB372561.1)	GGTCAGGCCTCAAGCTATCAA	CAGGGCCTCCGTCTCCTT	56	10	3
TIP2;1	*V. vinifera *aquaporin TIP2;1 (gb|EF364439.1)	GCATCATTGCACCCATTGC	GCCTGCAGCCAGGATGTT	59	6	1
CYSP	*V. vinifera *cysteine protease (gb|EU280160.1)	CCTCGCAGGAGGAGCACGAT	CCGGCGCAGGTTTGC	54	2	1
ERF4	*V. aestivalis *putative ethylene response factor 4 (gb|AY484580.1)	TCATCACTGCAACTCATCCA	TTACAATCTTCGGCCTCTGA	101	11	4
STS5	*V. vinifera *stilbene synthase5 (gb|AY670312.1)	CGCTCAAGGGAGGAAAGACA	AGCCAAACAAAACACCCCAATC	58	12	18
THX	thioredoxin x [Populus trichocarpa] (XP_002310066.1)	TGCTCAGGAATACGGGGACAGA	TCGCGGGTTTGCATCAT	61	-39	-2
SHM1	*A. thaliana *serine hydroxymethyl transferase 1 (ref|NM_119954.3)	TGTTCATCAGGTCAGCCAGTTT	TGCGTCGAATTGCAGCAAGAT	63	-2	-2
HypP	Hypothetical protein LOC100264849	TGCCCCTACCCTTGTGACA	GATCAAAATGGCTCATCGGAA	58	-5	-3
GLO	*V. pseudoreticulata *glyoxal oxidase (gb|D201181.1)	TCCCAACGCCGGTATAGC	ACCGTGCCGTAACGTGTGA	54	-5	-1
ClpP	*Carica papaya *ATP-dependent Clp protease proteolytic subunit (gb|DQ159405.1|)	GGGCGCCGGACAAGA	TTTGCAAATCATCCCTAATGGA	55	-2	-2

### Pathway enrichment analysis of DEGs

The PV affected biological pathways were evaluated by enrichment analysis of DEGs. Significantly enriched metabolic pathways and signal transduction pathways were identified. A total of 115 pathways were affected by up- and 107 were affected by down-regulated DEGs, respectively (additional file 2 and 3). DEGs with pathway annotation were listed according to enrichment priority (additional file 4 and 5). The first ten enriched pathways were reported in Table 5. Pathways with Q value < 0.05 are significantly enriched.

**Table 5:** List of first ten pathways for up- and downregulated EDGs.

Pathway term	Pathway ID	DEGs tested	P value	Q value
***Pathways for upregulated DEGs***				
Ribosome	ko03010	53 (4.36%)	0.0004	0.0406
Amino sugar and nucleotide sugar metabolism	ko00520	25 (2.06%)	0.0010	0.0563
Glycolysis/Gluconeogenesis	ko00010	28 (2.3%)	0.0043	0.1660
Biosynthesis of alkaloids derived from histidine and purine	ko01065	31 (2.55%)	0.0126	0.3636
Biosynthesis of alkaloids derived from ornithine, lysine and nicotinic acid	ko01064	35 (2.88%)	0.0207	0.4459
Starch and sucrose metabolism	ko00500	49 (4.03%)	0.0233	0.4459
Biosynthesis of alkaloids derived from shikimate pathway	ko01063	39 (3.21%)	0.0361	0.5868
N-Glycan biosynthesis	ko00510	10 (0.82%)	0.0528	0.5868
Fructose and mannose metabolism	ko00051	14 (1.15%)	0.0560	0.5868
Selenoamino acid metabolism	ko00450	11 (0.91%)	0.0587	0.5868
***Pathways for downregulated DEGs***				
Photosynthesis	ko00195	20 (3.14%)	9.9613e-06	0.0011
Photosynthesis - antenna proteins	ko00196	6 (0.94%)	4.2252e-05	0.0023
Folate biosynthesis	ko00790	5 (0.78%)	0.0002	0.0064
Nicotinate and nicotinamide metabolism	ko00760	5 (0.78%)	0.0007	0.0125
Fructose and mannose metabolism	ko00051	13 (2.04%)	0.0007	0.0125
Carbon fixation in photosynthetic organisms	ko00710	13 (2.04%)	0.0007	0.0125
Pyruvate metabolism	ko00620	14 (2.2%)	0.0014	0.0210
Polyketide sugar unit biosynthesis	ko00523	4 (0.63%)	0.0016	0.0210
Purine metabolism	ko00230	21 (3.3%)	0.0018	0.0215
Biosynthesis of alkaloids derived from histidine and purine	ko01065	21 (3.3%)	0.0025	0.0270

Ribosomal-associated proteins constituted the only significantly affected pathway for the upregulated DEGs (Q <0.05). Other non-significant enriched pathways with large number of upregulated DEGs included amino sugar and nucleotide sugar metabolism, starch and sucrose metabolism, secondary metabolism, plant hormone biosynthesis, and splicesome associated proteins. There were more significantly enriched pathways (10) for the downregulated DEGs, which were involved in photosynthesis, as well as metabolism of folate, nicotinate, nicotinamide, fructose, mannose, pyruvate, polyketide sugar unit, and purines, along with alkaloids from histidine and purines.

## Discussion

In this report Solexa sequencing technology, a high-throughput DNA sequencing approach, was utilized to estimate gene expression in libraries prepared from infected and control tissues. The results (Figure 2) provided estimates of gene expression as determined by the frequency that any given tag (representing a transcript) is sequenced. The data indicate that there is sufficient coverage depth to reach saturation, that is, a complete assessment of all transcripts present in the libraries. Theoretically, the rate of novel tag discovery should equal zero if all unique tags of the initial sample had been sequenced. However, this number might be slightly higher because new tags may be added due to the accumulation of sequencing errors as the size of the library increased [27]. Strict filtering and conservative matching allows recognition of erroneous tags, which are then disregarded. All of these precepts may contribute to a loss of substantial sequence information. However, loss of some data potentially made the results more conservative, revealing only robust and bona fide differences. Moreover, the total number of tags after stringent filtering was sufficient for annotation to the reference genes in the grape genome sequence. Theoretically, tags should be generated by *NlaIII *from the 3'-most ends of transcripts, but almost 50% of tags from other *NlaIII *sites were also generated in our result. Since only one tag could be generated in each transcript from any *NlaIII *site in a cDNA, these other *NlaIII *tags represented a given gene redundantly in the expression profile. This phenomenon accounts for the inflated number of unique tags generated (about 200,000) relative to that of the annotated grape genome (about 30,000). These other tags may also arise because of alternative splicing or incomplete enzyme digestion.

The results represent the first large-scale investigation of the gene expression in DM analysis of grapevine. Polesani et al [28] reported 804 transcripts identified in PV infected leaves of susceptible cultivar "Riesling" using cDNA-AFLP. Figueiredo et al [29] found 121 transcripts, representing 29 unique gene differentially expressed between two *V. vinifera *cultivars "Regent" and "Trincadeira" (resistant and susceptible to fungi, respectively) by cDNA microarray. In the current study, 15,249 putative genes were identified among the Solexa sequencing tags for the INF library and 14,549 for the CON library.

The steady-state transcript level for a set of selected genes was confirmed by Real-time RT-PCR. Although the differences in gene expression did not match the magnitude of those detected by Solexa-based sequencing method, the trends of up- and down- regulation were similar. The lower expression level detected by Real-time RT-PCR could be due to the difference of sensitivity between the two technologies. Solexa sequencing has been documented to be more sensitive for estimation of gene expression, especially for low-abundance transcripts compared to microarrays and Real-time RT-PCR [30]. The difference could also be attributed to different inoculation seasons and developmental stages of the grapevines. The materials used for the Solexa sequencing method were obtained from materials inoculated and harvested in September, while materials used for the Real-time RT- PCR analyses were obtained from plants inoculated and harvested in June.

Due to the sensitivity of Solexa sequencing technology, many rare transcripts were detected. Among 536 transcripts present predominantly (<2-20 fold) in the INF library, 89 were not detected in the CON library at all. These genes were predicted to be involved in many plant biological processes, including defense. For example, genes encoding cinnamyl alcohol dehydrogenase, lipase-like protein, glutathione synthetase, GDSL-motif lipase, ankyrin repeat family protein, serine hydrolase, proline-rich cell wall protein and multicopper oxidase were previously described as plant defense-related genes. Other rare transcripts detected by Solexa technology were predicted to function in signal transduction (protein kinase, calcium ion binding protein, wall-associated kinase), transport (type IIIa membrane protein, ATP binding protein, D-galactonate transporter, peptide transporter), transcription (ccaat-binding transcription factor, AP2/ERF domain-containing transcription factor, mutator-like transposase-like protein), and protein metabolism (ubiquitin-protein ligase, 50S ribosomal protein, S-locus-specific glycoprotein S13 precursor, Rab5-interacting protein). Two novel genes (nectar protein 1, vernalization-insensitive protein) and some genes encoding hypothetical proteins (LOC100244011, LOC100258240, LOC100249110) were also identified from the PV-induced rare DEGs. Among the 608 rare transcripts present more in CON than INF, 69 were not detected at all in the INF library. Most of these transcripts have predicated biological functions in growth regulation (growth regulator protein, A-type cyclin, auxin response factor 8), transport (ATP-binding cassette transporter, AWPM-19-like membrane family protein, copper-transporting atpase p-type), signal transduction (serine-threonine protein kinase, leucine-rich repeat family protein, calcium-binding EF hand family protein, calcium-dependent phospholipid binding ), and metabolism (galacturonosyltransferase 6, methylenetetrahydrofolate dehydrogenase, iron ion binding/oxidoreductase, trehalose-6-phosphate synthase, senescence-associated protein).

Pathway enrichment analysis revealed the most significantly affected pathways during the PV infection in "Zuoshan-1". It is not surprising that the "ribosome-related" pathway was the most affected for the DEGs more common in INF library. This finding implies that the grapevine utilizes new ribosomes or changes in ribosome components to help synthesize additional proteins, such as PR proteins, to protect itself from the pathogen attack. The second affected pathway was the "amino sugar and nucleotide sugar metabolism" pathway. In this pathway genes encoding chitinase were more prevalent in the INF than the CON library. In addition, genes required for cell wall biosynthesis were also affected, such as D-xylan synthase, UDP-glucose dehydrogenase, and UDP-glucose 4,6-dehydratase. These enzymes are involved in the interconversion of nucleotide sugars, and may regulate glycosylation patterns in response to pathogen, thereby linking signaling with primary metabolism and the dynamics of the extracellular matrix. The other noticeable pathways with a large amount of DEGs associated with PV infection were starch and sucrose metabolism, secondary metabolism, plant hormone biosynthesis, and splicesome-associated proteins. For DEGs less prevalent in infected vs. control libraries, there was significant enrichment for transcripts associated with photosynthesis. This result was similar to the reports of Polesani et al [28,31]. Photosystem I proteins (PsaA, PsaB, PsaC), photosystem II proteins (PsbB, PsbD, PsbO, PsbP, PsbS), cytochorme b6/f complex (PetD, PetN) and F-type ATPase (beta, alpha, delta, a, b) were all substantially lower in abundance in INF libraries compared to CON libraries. The reduction of photosynthesis was possibly due to the increase of invertase activity in nucleotide sugar metabolism pathway. Invertase would cleave sucrose into hexose sugars and their accumulation inhibits the Calvin cycle.

It was observed that 251 tags identified in INF library were homologous to the oomycete, indicating that they may belong to PV transcripts, predictably noting the presence of the pathogen. Many of these putative PV transcripts corresponded to genes involved in protein metabolism (16S, 18S, 26S, 28S and 60S ribosomal protein subunits) as a requirement for protein synthesis in the pathogen during the plant-pathogen interaction. Many housekeeping genes (alpha-tubulin, elongation factor 1 alpha, ubiquitin and heat shock protein 70) and genes related to immune response (spike 1 protein and cyclophilin) were also detected. Several PV transcripts showed similarity to enzymes involved in carbohydrate and amino acid metabolism (chlorophyll apoprotein, aspartate aminotransferase, glutamine synthetase and hyaluronoglucosaminidase-4), energy production (ATP synthase subunit B, glyceraldehyde-3-phosphate dehydrogenase, phosphoenolpyruvate carboxykinase and nitrate reductase), and cellular transport (transportin 1, K^+ ^channel protein and calmodulin).

### Transcripts more abundant in infected leaves

A set of transcripts were clearly more abundant in tissue arising after PV infection compared to control. This group possibly contains elements that confer resistance to the spread of the pathogen in "Zuoshan-1". Among these transcripts, those expressed at a relatively high level in infected tissue are of the most interest. These transcripts likely encode genes responding to the pathogen or genuine factors that underlie genetic resistance, which were broadly grouped into the following categories based on their known roles in other plant systems.

### Defense response genes

Among defense response genes, thaumatin-like protein [17], polygalacturonase-inhibiting protein (PGIP) [32,33], harpin-induced protein-related [34,35], glutaredoxin [36,37] and beta-glucosidase [38,39] have been widely studied in plant pathogen resistance. Thaumatin-like protein, like many other disease resistant proteins [40], is also induced by abiotic stresses, which may indicate existence of a crosstalk between pathogen and abiotic stresses. In this category, tobacco mosaic virus (TMV) response -related protein (+32 fold in INF vs CON) is associated with TMV attack and may also play an important role in DM resistance of grape.

### Transport

Three transcripts were associated with transport function. Multidrug resistance pump proteins (+121 fold in INF vs CON) and multidrug resistance ABC transporter (+25 fold in INF vs CON) are well known transporters in clinical study for bacteria infection of human [41]. Such transporters also have been isolated from plants, such as *Coptis japonica *[42]. They transport several compounds associated with multidrug (antibiotic) resistance which can inhibit pathogen infection in animal model [41,43]. Another gene identified to be transport related is mitochondrial dicarboxylate carrier protein (+38 fold in INF vs CON) which might be involved in the excretion of organic acids and rhizotoxic aluminum tolerance [44].

### Signal transduction

There were fourteen transcripts in our results associated with signal transduction. Two came from genes (GSVIVT00030628001, GSVIVT00030574001) encoding leucine-rich repeat receptor-like protein kinases which were more prevalent (145 and 20 fold) in the INF library than in control. Molecules that indicate the presence of pathogen (elicitors) activate host receptors and that rapidly generate an internal signal that triggers early defense responses [45]. Various signals presented in our results, including phytohormones like ABA and ethylene, as well as intracellular messengers like calcium, phosphoinositide and kinases, have been proposed to regulate plant responses in adverse environmental conditions and thus contribute to the coordination of plant stress physiology [46]. Transcripts representing three kinase-encoding genes (GSVIVT00030628001, GSVIVT00006178001, GSVIVT00019504001) were present 52-145 fold higher in INF than CON, and have been widely documented as signaling factors in many stresses [47-50] and senescence [51]. Four transcripts (GSVIVT00002706001, GSVIVT00020989001, GSVIVT00036549001, GSVIVT00002973001) were found to be more abundant (27 to 39 fold) in INF than CON, and were associated with calcium signaling pathway. All of these are also induced by senescence [52] and many stresses [53,54]. Nodulin-like protein (+23 fold in INF vs CON) induced in fungal pathogen treatment [55] and drought/heat combination stress [40] has been shown to be involved in salicylic acid (SA) signaling pathway [56]. A RING-H2 gene (+22 fold in INF vs CON) has demonstrated regulatory function in ABA signaling [57], drought tolerance [57], regulation of growth and defense responses against abiotic/biotic stresses [58]. Ethylene-regulated transcript 2 (ERT2) (+34 fold in INF vs CON) is involved in ethylene response 'circuit' including ethylene synthesis, perception, signal transduction and regulation of gene expression [59]. The PAR-1a (photoassimilate-responsive) protein (+22 fold in INF vs CON) is a serine/threonine kinase with diverse phosphorylation targets and has been reported to be induced by infection with potato virus Y [60,61].

### Transcription

Eleven transcripts associated with transcription were 21 to 60 fold more abundant in INF than CON libraries. Transcripts annotated as zinc-finger protein 1, DREB protein, AP2 domain class transcription factor, basic helix-loop-helix protein, CBF4(C-repeat binding factor 4), jasmonate ZIM domain 1, GRAS family transcription factor, and WRKY transcription factor 21 were all present at higher steady state levels in infected tissue. They have been documented to play important roles in responding to phytohormone stasis, pathogen attack and environmental stresses [62-69].

### Metabolism

#### Synthesis of the hormones

S-adenosyl-L-methionine (GSVIVT00024884001) and 9-cis-epoxycarotenoid dioxygenase 1(NCED1) (GSVIVT00000988001) are transcripts related to synthesis of plant hormones, and were found more frequently (97 and 62 fold, respectively) in the INF library. S-adenosyl-L-methionine is the precursor of ethylene [70] which participates in regulation of growth, development, and responses to stress and pathogen attack in plants [71]. NCED is an important enzyme in synthesizing the phytohormone ABA which plays a central role in responses to pathogen attack [72].

#### Protein metabolism

Twelve transcripts related to protein metabolism were more abundant in the INF library, 21 fold to 72 fold. Among them, ubiquitin-protein ligase (GSVIVT00028930001, GSVIVT00035825001), spotted leaf protein (GSVIVT00017518001, GSVIVT00028839001) and f-box family protein (GSVIVT00022245001, GSVIVT00009741001) were identified, and represent proteins involved in ubiquitination and subsequent degradation of target proteins. Aspartic proteinase nepenthesin-1 precursor (GSVIVT00032938001) is expressed at higher level in "Nipponbare" in response to phosphorus deficiency [73] and isolated from salt-stress wild rice "*Porteresia coarctata*" [74]. Protein phosphatase 2c (GSVIVT00024072001, GSVIVT00024235001, GSVIVT00001432001) regulates numerous ABA responses [75,76]. Nucleic acid binding proteins (GSVIVT00024387001) control genes expression in response to oxidative stress [77], ABA treatment [78] and abiotic stresses [79]. Exocyst subunit EXO70 family protein H4 (GSVIVT00023109001) has been shown to be involved in the exocytic pathway, which sorts newly synthesized proteins from the endoplasmic reticulum to their final destination at the lysosome, vacuole or plasma membrane [80].

#### Secondary metabolism

This subcategory contained 4 genes, including a higher level of tropinone reductase (GSVIVT00018424001, +48 fold in INF vs CON) transcript in infected leaves, consistent with previous reports showing it to be more abundant after pathogen infection [81]. Isoflavone reductase-like protein 3 (GSVIVT00019233001, +31 fold in INF vs CON) also has a potential pathogen resistance role because it is involved in biosynthesis of isoflavonoid phytoalexins [82], an important product in resistance to pathogen infection [83,84]. UDP-glucose glucosyltransferase (GSVIVT00002450001, + 24 fold in INF vs CON) and galactinol synthase (GSVIVT00019669001, + 24 fold in INF vs CON) are reported to be induced by abiotic stresses [85,86].

#### Cell wall organization

Three genes were classified into this subcategory. Cellulose synthase-like D1 (GSVIVT00014029001, + 31 fold in INF vs CON) and beta-expansin 1a precursor (GSVIVT00036225001, + 27 fold in INF vs CON) contribute to cell wall synthesis and modification [87,88]. The wound-induced protein (WIN2) (GSVIVT00007452001, + 26 fold in INF vs CON) with anti-fungal activity [89] possesses a domain that binds PAMP (pathogen-associated molecular patterns) elicitors (e.g., chitin) [90] and is induced in response to pathogen. In addition, other highly expressed metabolic genes in the INF samples were glucose-1-phosphate adenylyltransferase (GSVIVT00036349001, + 24 fold in INF vs CON), cytochrome P450 (GSVIVT00014730001, + 70 fold in INF vs CON) and serine acetyltransferase (GSVIVT00007984001, + 30 fold in INF vs CON). These transcripts are related to carbohydrate metabolism, photosynthesis and cysteine synthesis. Cysteine synthesis has reported to respond to oxidative stress by calcium signaling [91].

Even though most of these genes have been reported to be biotic or abiotic stresses related, seven high expressed genes in the infected leaves have not been previously reported being associated with stress. They were noted as protein-binding protein (GSVIVT00024408001, + 87 fold in INF vs CON), ATPP2-A2 (*Arabidopsis thaliana *phloem protein 2-A2) (GSVIVT00023009001, + 56 fold in INF vs CON), putative integral membrane protein (GSVIVT00014704001, + 51 fold in INF vs CON), putative phosphate-induced protein (GSVIVT00015203001, + 229; GSVIVT00015200001, +37 fold in INF vs CON), ATP-dependent DNA helicase (GSVIVT00016166001, +36 fold in INF vs CON), CW14 (GSVIVT00020834001, +23 fold in INF vs CON), and a hypothetical protein (GSVIVT00017533001, +20 fold in INF vs CON).

### Transcripts less abundant in infected leaves

The most striking functions for transcripts less abundant in infected tissue were those associated with metabolism and defense response to pathogen attack. Fifteen DEGs were detected to be less prevalent in the INF libraries more than 20 fold compared to CON, most of which, such as (-)-germacrene D synthase [92], non-specific lipid transfer protein [93], major histocompatibility complex [94], thioredoxin [95], beta-cyano-alanine synthase [96], expansin [97] and UDP-glucosyltransferase [98] are reported to be positively associated with plant defense responses to pathogen attack. However, our data indicated that the expression level of these transcripts was lower in infected tissues.

Another two transcripts that were less prevalent in infected tissue (GSVIVT00014727001, -35 fold in INF vs CON; GSVIVT00014725001, -41 in INF vs CON) belong to cytochrome P450 family with oxidative function. Interestingly, a novel gene encoding male sterility-related protein was also identified in this group, and its function associated with DM response has not been clarified.

## Conclusions

Solexa-based sequencing can be used for analyzing variation in gene expression between two samples. The gene expression level in "Zuoshan-1" leaves infected with PV changed significantly in comparison with control leaves. Analysis of differentially-expressed genes involved in the pathogen infection allows delineation of candidate genes potentially relevant to DM resistance in grapevines.

## Methods

### Plants material and pathogen infection

One-year-old, certified virus-free seedlings of "Zuoshan-1" were grown and maintained in the greenhouse under a 16-h light/8-h dark photoperiod at 25°C, 85% relative humidity. Control plants were maintained under the same conditions. *P. viticola *was collected from sporulated field leaves and used for the artificial inoculations of surface-sterilized leaves. Infections were conducted by dipping the fourth grapevine leaves in a suspension of 10,000 sporangia per ml pure water. The leaves were covered with plastic bags for one night to ensure high humidity. The fourth unfolded leaf from the shoot apex was harvested from each of three vines, and the three leaves were combined to represent one replicate. Three independent replicates were collected for each sample. Infected leaves were collected every 24 h for 9 days. Control samples were harvested from water-treated leaves incubated under the same conditions.

### Preparation of Digital Expression Libraries

Samples from infected leaves from 4 d to 8 d were pooled for RNA isolation and library construction. Comparable control leaves were treated identically and in parallel. Total RNA was isolated from the leaf mixture using a modification of the CTAB method as presented by Murray and Thompson [99]. Sequence tag preparation was done with the Digital Gene Expression Tag Profiling Kit (Illumina Inc; San Diego, CA, USA) according to the manufacturer's protocol (version 2.1B). Six micrograms of total RNA was extracted and mRNA was purified using biotin-Oligo (dT) magnetic bead adsorption. First- and second-strand cDNA synthesis was performed after the RNA was bound to the beads. While on the beads, double strand cDNA was digested with *NlaIII *endonuclease to produce a bead-bound cDNA fragment containing sequence from the 3'-most CATG to the poly (A)-tail. These 3' cDNA fragments were purified using magnetic bead precipitation and the Illumina adapter 1 (GEX adapter 1) was added to new 5' end. The junction of Illumina adapter 1 and CATG site was recognized by *MmeI*, which is a Type I endonuclease (with separated recognition sites and digestion sites). The enzyme cuts 17 bp downstream of the CATG site, producing 17 bp cDNA sequence tags with adapter 1. After removing 3' fragments with magnetic bead precipitation, the Illumina adapter 2 (GEX adapter 2) was ligated to 3' end of the cDNA tag. These cDNA fragments represented the tag library.

### Solexa sequencing

Sequencing was performed by "HuaDa Gene" [100] with the method of sequencing by synthesis. A PCR amplification with 15 cycles using Phusion polymerase (Finnzymes, Espoo, Finland) was performed with primers complementary to the adapter sequences to enrich the samples for the desired fragments. The resulting 85 base strips were purified by 6% TBE PAGE Gel electrophoresis. These strips were then digested, and the single-chain molecules were fixed onto the Solexa Sequencing Chip (flow cell). Each molecule grew into a single-molecule cluster sequencing template through in situ amplification. Four color-labeled nucleotides were added, and sequencing was performed with the method of sequencing by synthesis. Image analysis and basecalling were performed using the Illumina Pipeline, and cDNA sequence tags were revealed after purity filtering. The tags passing initial quality tests were sorted and counted. Each tunnel generates millions of raw reads with sequencing length of 35 bp (target tags plus 3'adaptor). Each molecule in the library represented a single tag derived from a single transcript.

### Sequence annotation

"Clean Tags" were obtained by filtering off adaptor-only tags and low-quality tags (containing ambiguous bases). Comparison of the sequences by blastn was carried out using the following databases: NCBI [101], Genoscope Grape Genome database [25] and VBI Microbial Database [26]. All clean tags were annotated based on grape reference genes. For conservative and precise annotation, only sequences with perfect homology or 1 nt mismatch were considered further. The number of annotated clean tags for each gene was calculated and then normalized to TPM (number of transcripts per million clean tags) [30,102]. Sequences were manually assigned to functional categories based on the analysis of scientific literature.

### Identification of differentially expressed genes (DEGs)

A rigorous algorithm to identify differentially expressed genes between two samples was developed [103]. P value was used to test differential transcript accumulation. In the formula below the total clean tag number of the CON library is noted as N1, and total clean tag number of INF library as N2; gene A holds x tags in CON and y tags in INF library. The probability of gene A expressed equally between two samples can be calculated with:

$$\text{P}(\text{y}|\text{x})={\left(\frac{N2}{N1}\right)}^{\text{y}}\frac{(\text{x}+\text{y})!}{\text{x}!\text{y}!{\left(1+\frac{N_{2}}{N_{1}}\right)}^{\left(\text{x}+\text{y}+1\right)}}$$

FDR (False Discovery Rate) was applied to determine the threshold of P Value in multiple tests and analyses [104]. An "FDR < 0.001 and the absolute value of log2Ratio ≥ 1" was used as the threshold to judge the significance of gene expression difference.

### Real-time RT-PCR analysis

Samples were prepared using the same method mentioned above and total RNA was isolated from the leaf mixture. Experiments were carried out on three independent biological replicates each containing three technical replicates. First-strand cDNA was synthesized from 650 ng DNase (Promega, Madison, Wisconsin, USA) -treated total RNA using "ImProm-II TM Reverse Transcriptase" (Promega, Madison, Wisconsin, USA) and diluted 20 fold as template. Specific primer pairs of twelve randomly selected genes were designed (Table 4) using Primer Express 3.0 and tested by Real-time RT-PCR. Primers specific for *V. vinifera *actin (Forward: AATGTGCCTGCCATGTATGT; Reverse: TCACACCATCACCAGAATCC) were used for the normalization of reactions. Experiments were carried out using Power SYBR Green PCR Master Mix (Applied Biosystems, Warrington, UK) in a StepOne™ Real-Time PCR System (Applied Biosystems). The reaction volume was 20 μl, including 10 μl Power SYBR Green PCR master mix, 0.9 μl 10 mM primer, 2.0 μl cDNA sample and 6.20 μl dH2O. The following thermal cycling profile was used: 95°C 10 min; 40 cycles of 95°C for 15 s, 59°C for 1 min; 95°C for 15 s, 60°C for 1 min, 95°C for 15 s. Data were analyzed using StepOne™ Software Version 2.0 (Applied Biosystems). Actin expression was used as an internal control to normalize all data. The fold change in mRNA expression was estimated using threshold cycles, by the ΔΔCT method [105].

### Pathway Enrichment Analysis of DEGs

Pathway enrichment analysis based on KEGG [106] was used to identify significantly enriched metabolic pathways or signal transduction pathways in differentially-expressed genes comparing with the whole genome background. The calculating formula is:

$$\text{P}=1-\sum_{i=0}^{m-1}\frac{\left(\begin{array}{c} M\\ i \end{array}\right)\left(\begin{array}{c} N-M\\ n-i \end{array}\right)}{\left(\begin{array}{c} N\\ n \end{array}\right)}$$

where N is the number of all genes that with KEGG annotation, n is the number of DEGs in N, M is the number of all genes annotated to specific pathways, and m is number of DEGs in M. Q value was used for determining the threshold of P Value in multiple test and analysis [107]. Pathways with Q value < 0.05 are significantly enriched in DEGs.

## Abbreviations

AFLP: Amplified Fragment Length Polymorphism; BLAST: Basic Local Alignment Search Tool; cDNA: Complementary DNA; CTAB: Hexadecyltrimethylammonium bromide; DEGs: differentially expressed transcripts; NCBI: National Center for Biotechnology Information.

## Authors' contributions

JW and YLZ carried out the plant material preparation, PV infection, RNA extraction, preparation of digital expression libraries, sequence analysis, and contributed to data interpretation and manuscript writing. HQZ participated in PV infection and RNA extraction. HH contributed to sequence analysis. KMF participated in data interpretation and manuscript modification. JL conceived the study, led the experiment design and coordinated all the research activities, contributed to interpretation of the data, manuscript writing and modification. All authors read and approved the final manuscript.

## Supplementary Material

Additional file 1 **Complete list of transcripts attributed to *P. viticola***. Click here for file

Additional file 2 **Complete list of involved pathways for upregualted DEGs**. Pathways with Q value < 0.05 are significantly enriched for upregulated DEGs. Click here for file

Additional file 3 **Complete list of involved pathways for downregualted DEGs**. Pathways with Q value < 0.05 are significantly enriched for downregulated DEGs. Click here for file

Additional file 4 **List of "Zuoshan-1" transcripts upregulated for at least 2 fold in INF library**. Two fold and more upregualted genes with pathway annotation in INF library were listed in different categories. Click here for file

Additional file 5 **List of "Zuoshan-1" transcripts downregulated for at least 2 fold in INF library**. Two fold and more downregualted genes with pathway annotation in INF library were listed in different categories. Click here for file

## References

[B1] Pearson RC Goheen AC Compendium of Grape Diseases 1988 APS Press

[B2] Spencer DM The Downy mildews 1981 London: Academic Press

[B3] Kiefer B Riemann M Buche C Kassemeyer HH Nick P The host guides morphogenesis and stomatal targeting in the grapevine pathogen *Plasmopara viticola* Planta 2002 215 387 393 10.1007/s00425-002-0760-2 12111219

[B4] Staudt G Kassemeyer HH Evaluation of downy mildew resistance in various accessions of wild *Vitis *species Vitis 1995 34 225 228

[B5] Brown MV Morre JN Fenn P McNew RW Evaluation of grape germplasm for downy mildew resistance Fruit Varieties Journal 1999 53 22 29

[B6] Olmo HP *Vinifera *x *rotundifolia *hybrids as wines grapes Am J Enol Vitic 1971 22 87 91

[B7] Dai GH Andary C Mondolot-Cosson L Boubals D Histochemical studies on the interaction between three species of grapevine, *Vitis vinifera*, *V. rupestris *and *V. rotundifolia *and the downy mildew fungus, *Plasmopara viticola* Physiol Mol Plant Pathol 1995 46 177 188 10.1006/pmpp.1995.1014

[B8] Unger S Buche C Boso S Kassemeyer HH The Course of Colonization of Two Different *Vitis *Genotypes by *Plasmopara viticola *Indicates Compatible and Incompatible Host-Pathogen Interactions Phytopathology 2007 97 780 786 10.1094/PHYTO-97-7-0780 18943926

[B9] Diez-Navajas AM Wiedemann-Merdinoglu S Greif C Merdinoglu D Nonhost versus host resistance to the grapevine downy mildew, *Plasmopara viticola*, studied at the tissue level Phytopathology 2008 98 776 780 10.1094/PHYTO-98-7-0776 18943253

[B10] Merdinoglu D Wiedemann-Merdinoglu S Coste P Dumas V Haetty S Butterlin G Greif C Genetic Analysis of Downy Mildew Resistance Derived from *Muscadinia rotundifolia* Acta Hort 2003 603 451 456

[B11] Henanff GL Heitz T Mestre P Mutterer J Walter B Chong J Characterization of *Vitis vinifera *NPR1 homologs involved in the regulation of Pathogenesis-Related gene expression BMC Plant Biology 2009 9 54 67 10.1186/1471-2229-9-54 19432948 PMC2686700

[B12] Kortekamp A Expression analysis of defence-related genes in grapevine leaves after inoculation with a host and a non-host pathogen Plant Physiol Biochem 2006 44 58 67 10.1016/j.plaphy.2006.01.008 16531058

[B13] Slaughter AR Hamiduzzaman MM Gindro K Neuhaus JM Mauch-Mani B Beta-aminobutyric acid-induced resistance in grapevine against downy mildew: involvement of pterostilbene Eur J Plant Pathol 2008 122 185 195 10.1007/s10658-008-9285-2

[B14] Kini KR Vasanthi NS Shetty HS Induction of beta-1,3-glucanase in Seedlings of Pearl Millet in Response to Infection by *Sclerospora graminicola* European Journal of Plant Pathology 2000 106 267 274 10.1023/A:1008771124782

[B15] Richter H Pezet R Viret O Gindro K Characterization of 3 new partial stilbene synthase genes out of over 20 expressed in *Vitis vinifera *during the Interaction with *Plasmopara viticola* Physiological and Molecular Plant Pathology 2006 67 248 260 10.1016/j.pmpp.2006.03.001

[B16] Nagarathna KC Shetty SA Shetty HS Phenylalanine Ammonia Lyase Activity in Pearl Millet Seedlings and its Relation to Downy Mildew Disease Resistance Journal of Experimental Botany 1993 44 1291 1296 10.1093/jxb/44.8.1291

[B17] Punja ZK Genetic engineering of plants to enhance resistance to fungal pathogens-a review of progress and future prospects Plant Pathol 2001 23 216 235

[B18] Yamamoto T Iketani H Ieki H Nishizawa Y Notsuka K Hibi T Hayashi T Matsuta N Transgenic grapevine plants expressing a rice chitinase with enhanced resistance to fungal pathogens Plant Cell Reports 2000 19 639 646 10.1007/s002999900174 30754799

[B19] Vidal JR Kikkert JR Wallace PG Reisch BI High-efficiency biolistic co-transformation and regeneration of 'Chardonnay' (*Vitis vinifera *L.) containing *npt-II *and antimicrobial peptide genes Plant Cell Rep 2003 22 252 260 10.1007/s00299-003-0682-x 12908080

[B20] Bornhoff BA Harst M Zyprian E Topfer R Transgenic plants of *Vitis vinifera *cv. Seyval blanc Plant Cell Rep 2005 24 433 438 10.1007/s00299-005-0959-3 15812658

[B21] Fan CH Pu N Wang XP Wang YJ Fang L Xu WR Zhang JX *Agrobacterium*-mediated genetic transformation of grapevine (*Vitis vinifera *L.) with a novel stilbene synthase gene from Chinese wild *Vitis pseudoreticulata* Plant Cell Tiss Organ Cult 2008 92 197 206 10.1007/s11240-007-9324-2

[B22] Angarawai II Kadams AM Bello D Gene Effects Controlling Heritability of Downy Mildew Resistance in Nigerian Elite Pearl Millet Lines World Journal of Agricultural Sciences 2008 4 545 549

[B23] Zyprian E Welter LJ Akkurt M Ebert S Salakhutdinov I Göktürk-Baydar N Eibach R Töpfer R Candidate genes mapping and comparative QTL analysis for powdery and downy mildew resistance in grape Acta Hort 2009 827 535 538

[B24] Li X Shen Y Ge Y Zang P Ai J Jin S Study for evaluating infecting - disease property on *plasmopara viticola *to the germplasm resources of *vitis amurensis *rupr Special Wild Economic Animal and Plant Research 1999 2 10 13

[B25] Genoscope Grape Genome database http://www.cns.fr/spip/Vitis-ninifera-e.html

[B26] VBI Microbial Database http://phytophthora.vbi.vt.edu/

[B27] Keime C Semon M Mouchiroud D Duret L Gandrillon O Unexpected observations after mapping LongSAGE tags to the human genome BMC Bioinformatics 2007 8 154 10.1186/1471-2105-8-154 17504516 PMC1884178

[B28] Polesani M Desario F Ferrarini A Zamboni A Pezzotti M Kortekamp A Polverari A cDNA-AFLP analysis of plant and pathogen genes expressed in grapevine infected with *Plasmopara viticola* BMC Genomics 2008 9 142 156 10.1186/1471-2164-9-142 18366764 PMC2292706

[B29] Figueiredo A Fortes AM Ferreira S Sebastiana M Choi YH Sousa L Acioli-Santos B Pessoa F Verpoorte R Pais MS Transcriptional and metabolic profiling of grape (*Vitis vinifera *L.) leaves unravel possible innate resistance against pathogenic fungi J Exp Bot 2008 59 3371 3381 10.1093/jxb/ern187 18648103

[B30] t Hoen PA Ariyurek Y Thygesen HH Vreugdenhil E Vossen RH de Menezes RX Boer JM van Ommen GJ den Dunnen JT Deep sequencing-based expression analysis shows major advances in robustness, resolution and inter-lab portability over five microarray platforms Nucleic Acids Res 2008 36 e141 10.1093/nar/gkn705 18927111 PMC2588528

[B31] Polesani M Bortesi L Ferrarini A Zamboni A Fasoli M Zadra C Lovato A Pezzotti M Delledonne M Polverari A General and species-specific transcriptional responses to downy mildew infection in a susceptible (*Vitis vinifera*) and a resistant (*V. riparia*) grapevine species BMC Genomics 2010 11 117 10.1186/1471-2164-11-117 20167053 PMC2831845

[B32] Di Matteo A Bonivento D Tsernoglou D Federici L Cervone F Polygalacturonase-inhibiting protein (PGIP) in plant defence: a structural view Phytochemistry 2006 67 528 533 10.1016/j.phytochem.2005.12.025 16458942

[B33] Joubert DA Kars I Wagemakers L Bergmann C Kemp G Vivier MA van Kan JA A polygalacturonase-inhibiting protein from grapevine reduces the symptoms of the endopolygalacturonase BcPG2 from *Botrytis cinerea *in *Nicotiana **benthamiana *leaves without any evidence for in vitro interaction Mol Plant Microbe Interact 2007 20 392 402 10.1094/MPMI-20-4-0392 17427809

[B34] Ditt RF Kerr KF de Figueiredo P Delrow J Comai L Nester EW The *Arabidopsis thaliana *transcriptome in response to *Agrobacterium tumefaciens* Mol Plant Microbe Interact 2006 19 665 681 10.1094/MPMI-19-0665 16776300

[B35] Jolivet K Grenier E Bouchet JP Esquibet M Kerlan MC Caromel B Mugniery D Lefebvre V Identification of plant genes regulated in resistant potato *Solanum sparsipilum *during the early stages of infection by *Globodera pallida* Genome 2007 50 422 427 10.1139/G07-015 17546100

[B36] Ndamukong I Abdallat AA Thurow C Fode B Zander M Weigel R Gatz C SA-inducible *Arabidopsis *glutaredoxin interacts with TGA factors and suppresses JA-responsive PDF1.2 transcription Plant J 2007 50 128 139 10.1111/j.1365-313X.2007.03039.x 17397508

[B37] Wang Z Xing S Birkenbihl RP Zachgo S Conserved functions of *Arabidopsis *and rice CC-type glutaredoxins in flower development and pathogen response Mol Plant 2009 2 323 335 10.1093/mp/ssn078 19825617

[B38] Beffa RS Neuhaus JM Meins F Jr Physiological compensation in antisense transformants: specific induction of an "ersatz" glucan endo-1,3-beta-glucosidase in plants infected with necrotizing viruses Proc Natl Acad Sci USA 1993 90 8792 8796 10.1073/pnas.90.19.8792 8415609 PMC47446

[B39] Mattiacci L Dicke M Posthumus MA beta-Glucosidase: an elicitor of herbivore-induced plant odor that attracts host-searching parasitic wasps Proc Natl Acad Sci USA 1995 92 2036 2040 10.1073/pnas.92.6.2036 11607516 PMC42418

[B40] Rizhsky L Liang H Shuman J Shulaev V Davletova S Mittler R When defense pathways collide. The response of *Arabidopsis *to a combination of drought and heat stress Plant Physiol 2004 134 1683 1696 10.1104/pp.103.033431 15047901 PMC419842

[B41] Piddock LJ Multidrug-resistance efflux pumps - not just for resistance Nat Rev Microbiol 2006 4 629 636 10.1038/nrmicro1464 16845433

[B42] Shitan N Bazin I Dan K Obata K Kigawa K Ueda K Sato F Forestier C Yazaki K Involvement of CjMDR1, a plant multidrug-resistance-type ATP-binding cassette protein, in alkaloid transport in *Coptis japonica* Proc Natl Acad Sci USA 2003 100 751 756 10.1073/pnas.0134257100 12524452 PMC141068

[B43] Morschhauser J Regulation of multidrug resistance in pathogenic fungi Fungal Genet Biol 47 94 106 10.1016/j.fgb.2009.08.002 19665571

[B44] Deng W Luo K Li Z Yang Y Molecular cloning and characterization of a mitochondrial dicarboxylate/tricarboxylate transporter gene in *Citrus junos *response to aluminum stress Mitochondrial DNA 2008 19 376 384 19462511

[B45] Blumwald E Aharon GS Lam BCH Early signal transduction pathways in plant-pathogen interactions Trends in Plant Science 1998 3 342 346 10.1016/S1360-1385(98)01289-8

[B46] Zhu JK Salt and drought stress signal transduction in plants Annu Rev Plant Biol 2002 53 247 273 10.1146/annurev.arplant.53.091401.143329 12221975 PMC3128348

[B47] Xiong L Lee B Ishitani M Lee H Zhang C Zhu JK FIERY1 encoding an inositol polyphosphate 1-phosphatase is a negative regulator of abscisic acid and stress signaling in *Arabidopsis* Genes Dev 2001 15 1971 1984 10.1101/gad.891901 11485991 PMC312749

[B48] Tang D Christiansen KM Innes RW Regulation of plant disease resistance, stress responses, cell death, and ethylene signaling in *Arabidopsis *by the EDR1 protein kinase Plant Physiol 2005 138 1018 1026 10.1104/pp.105.060400 15894742 PMC1150416

[B49] You MK Oh SI Ok SH Cho SK Shin HY Jeung JU Shin JS Identification of putative MAPK kinases in *Oryza minuta *and *O. sativa *responsive to biotic stresses Mol Cells 2007 23 108 114 17464219

[B50] Pitzschke A Schikora A Hirt H MAPK cascade signalling networks in plant defence Curr Opin Plant Biol 2009 12 421 426 10.1016/j.pbi.2009.06.008 19608449

[B51] Zhou C Cai Z Guo Y Gan S An *arabidopsis *mitogen-activated protein kinase cascade, MKK9-MPK6, plays a role in leaf senescence Plant Physiol 2009 150 167 177 10.1104/pp.108.133439 19251906 PMC2675715

[B52] Espinoza C Medina C Somerville S Arce-Johnson P Senescence-associated genes induced during compatible viral interactions with grapevine and *Arabidopsis* J Exp Bot 2007 58 3197 3212 10.1093/jxb/erm165 17761729

[B53] Knight H Calcium signaling during abiotic stress in plants Int Rev Cytol 2000 195 269 324 full_text 10603578 10.1016/s0074-7696(08)62707-2

[B54] Li A Wang X Leseberg CH Jia J Mao L Biotic and abiotic stress responses through calcium-dependent protein kinase (CDPK) signaling in wheat (*Triticum aestivum *L.) Plant Signal Behav 2008 3 654 666 19704816 10.4161/psb.3.9.5757 PMC2634547

[B55] Siciliano V Genre A Balestrini R Dewit PJ Bonfante P Pre-Penetration Apparatus Formation During AM Infection is Associated With a Specific Transcriptome Response in Epidermal Cells Plant Signal Behav 2007 2 533 545 19704551 10.4161/psb.2.6.4745 PMC2634361

[B56] Peleg-Grossman S Golani Y Kaye Y Melamed-Book N Levine A NPR1 protein regulates pathogenic and symbiotic interactions between *Rhizobium *and legumes and non-legumes PLoS One 2009 4 e8399 10.1371/journal.pone.0008399 20027302 PMC2793007

[B57] Ko JH Yang SH Han KH Upregulation of an *Arabidopsis *RING-H2 gene, *XERICO*, confers drought tolerance through increased abscisic acid biosynthesis Plant J 2006 47 343 355 10.1111/j.1365-313X.2006.02782.x 16792696

[B58] Liu H Zhang H Yang Y Li G Wang X Basnayake BM Li D Song F Functional analysis reveals pleiotropic effects of rice RING-H2 finger protein gene *OsBIRF1 *on regulation of growth and defense responses against abiotic and biotic stresses Plant Mol Biol 2008 68 17 30 10.1007/s11103-008-9349-x 18496756

[B59] Zhong GY Burns JK Profiling ethylene-regulated gene expression in *Arabidopsis thaliana *by microarray analysis Plant Mol Biol 2003 53 117 131 10.1023/B:PLAN.0000009270.81977.ef 14756311

[B60] Guo S Kemphues KJ *par-1*, a gene required for establishing polarity in *C. elegans *embryos, encodes a putative Ser/Thr kinase that is asymmetrically distributed Cell 1995 81 611 620 10.1016/0092-8674(95)90082-9 7758115

[B61] Herbers K Monke G Badur R Sonnewald U A simplified procedure for the subtractive cDNA cloning of photoassimilate-responding genes: isolation of cDNAs encoding a new class of pathogenesis-related proteins Plant Mol Biol 1995 29 1027 1038 10.1007/BF00014975 8555446 PMC7088993

[B62] Takatsuji H Zinc-finger transcription factors in plants Cell Mol Life Sci 1998 54 582 596 10.1007/s000180050186 9676577 PMC11147231

[B63] Agarwal PK Agarwal P Reddy MK Sopory SK Role of DREB transcription factors in abiotic and biotic stress tolerance in plants Plant Cell Rep 2006 25 1263 1274 10.1007/s00299-006-0204-8 16858552

[B64] Pre M Atallah M Champion A De Vos M Pieterse CM Memelink J The AP2/ERF domain transcription factor ORA59 integrates jasmonic acid and ethylene signals in plant defense Plant Physiol 2008 147 1347 1357 10.1104/pp.108.117523 18467450 PMC2442530

[B65] Duek PD Fankhauser C bHLH class transcription factors take centre stage in phytochrome signalling Trends Plant Sci 2005 10 51 54 10.1016/j.tplants.2004.12.005 15708340

[B66] Haake V Cook D Riechmann JL Pineda O Thomashow MF Zhang JZ Transcription factor CBF4 is a regulator of drought adaptation in *Arabidopsis* Plant Physiol 2002 130 639 648 10.1104/pp.006478 12376631 PMC166593

[B67] Chung HS Koo AJ Gao X Jayanty S Thines B Jones AD Howe GA Regulation and function of *Arabidopsis *JASMONATE ZIM-domain genes in response to wounding and herbivory Plant Physiol 2008 146 952 964 10.1104/pp.107.115691 18223147 PMC2259048

[B68] Smit P Raedts J Portyanko V Debelle F Gough C Bisseling T Geurts R NSP1 of the GRAS protein family is essential for *rhizobial *Nod factor-induced transcription Science 2005 308 1789 1791 10.1126/science.1111025 15961669

[B69] Eulgem T Rushton PJ Robatzek S Somssich IE The WRKY superfamily of plant transcription factors Trends Plant Sci 2000 5 199 206 10.1016/S1360-1385(00)01600-9 10785665

[B70] Roje S S-Adenosyl-L-methionine: beyond the universal methyl group donor Phytochemistry 2006 67 1686 1698 10.1016/j.phytochem.2006.04.019 16766004

[B71] Bleecker AB Kende H Ethylene: a gaseous signal molecule in plants Annu Rev Cell Dev Biol 2000 16 1 18 10.1146/annurev.cellbio.16.1.1 11031228

[B72] de Torres-Zabala M Truman W Bennett MH Lafforgue G Mansfield JW Rodriguez Egea P Bogre L Grant M *Pseudomonas syringae *pv. tomato hijacks the *Arabidopsis *abscisic acid signalling pathway to cause disease EMBO J 2007 26 1434 1443 10.1038/sj.emboj.7601575 17304219 PMC1817624

[B73] Pariasca-Tanaka J Satoh K Rose T Mauleon R Wissuwa M Stress Response Versus Stress Tolerance: A Transcriptome Analysis of Two Rice Lines Contrasting in Tolerance to Phosphorus Deficiency Rice 2009 2 167 185 10.1007/s12284-009-9032-0

[B74] Sengupta S Majumder AL *Porteresia coarctata *(Roxb.) Tateoka, a wild rice: a potential model for studying salt-stress biology in rice Plant Cell Environ 2010 33 526 542 10.1111/j.1365-3040.2009.02054.x 19843254

[B75] Gosti F Beaudoin N Serizet C Webb AA Vartanian N Giraudat J ABI1 protein phosphatase 2C is a negative regulator of abscisic acid signaling Plant Cell 1999 11 1897 1910 10.1105/tpc.11.10.1897 10521520 PMC144098

[B76] Vlad F Rubio S Rodrigues A Sirichandra C Belin C Robert N Leung J Rodriguez PL Lauriere C Merlot S Protein phosphatases 2C regulate the activation of the Snf1-related kinase OST1 by abscisic acid in *Arabidopsis* Plant Cell 2009 21 3170 3184 10.1105/tpc.109.069179 19855047 PMC2782292

[B77] Rodriguez-Gabriel MA Burns G McDonald WH Martin V Yates JR Bahler J Russell P RNA-binding protein Csx1 mediates global control of gene expression in response to oxidative stress EMBO J 2003 22 6256 6266 10.1093/emboj/cdg597 14633985 PMC291838

[B78] Razem FA El-Kereamy A Abrams SR Hill RD The RNA-binding protein FCA is an abscisic acid receptor Nature 2006 439 290 294 10.1038/nature04373 16421562

[B79] Kim JS Jung HJ Lee HJ Kim KA Goh CH Woo Y Oh SH Han YS Kang H Glycine-rich RNA-binding protein 7 affects abiotic stress responses by regulating stomata opening and closing in *Arabidopsis thaliana* Plant J 2008 55 455 466 10.1111/j.1365-313X.2008.03518.x 18410480

[B80] Dong G Hutagalung AH Fu C Novick P Reinisch KM The structures of exocyst subunit Exo70p and the Exo84p C-terminal domains reveal a common motif Nat Struct Mol Biol 2005 12 1094 1100 10.1038/nsmb1017 16249794

[B81] Espinoza C Vega A Medina C Schlauch K Cramer G Arce-Johnson P Gene expression associated with compatible viral diseases in grapevine cultivars Funct Integr Genomics 2007 7 95 110 10.1007/s10142-006-0031-6 16775684

[B82] Kim ST Cho KS Kim SG Kang SY Kang KY A rice isoflavone reductase-like gene, *OsIRL*, is induced by rice blast fungal elicitor Mol Cells 2003 16 224 231 14651265

[B83] Prasanna TB Vairamani M Kasbekar DP Effects of pisatin on *Dictyostelium **discoideum*: its relationship to inducible resistance to nystatin and extension to other isoflavonoid phytoalexins Arch Microbiol 1998 170 309 312 10.1007/s002030050647 9732446

[B84] He XZ Dixon RA Genetic manipulation of isoflavone 7-O-methyltransferase enhances biosynthesis of 4'-O-methylated isoflavonoid phytoalexins and disease resistance in alfalfa Plant Cell 2000 12 1689 1702 10.1105/tpc.12.9.1689 11006341 PMC149079

[B85] Gachon CM Langlois-Meurinne M Saindrenan P Plant secondary metabolism glycosyltransferases: the emerging functional analysis Trends Plant Sci 2005 10 542 549 10.1016/j.tplants.2005.09.007 16214386

[B86] Nishizawa-Yokoi A Yabuta Y Shigeoka S The contribution of carbohydrates including raffinose family oligosaccharides and sugar alcohols to protection of plant cells from oxidative damage Plant Signal Behav 2008 3 1016 1018 19704439 10.4161/psb.6738 PMC2633762

[B87] Hazen SP Scott-Craig JS Walton JD Cellulose synthase-like genes of rice Plant Physiol 2002 128 336 340 10.1104/pp.010875 11842136 PMC1540205

[B88] Zheng J Fu J Gou M Huai J Liu Y Jian M Huang Q Guo X Dong Z Wang H Genome-wide transcriptome analysis of two maize inbred lines under drought stress Plant Mol Biol 72 407 421 10.1007/s11103-009-9579-6 19953304

[B89] Stanford A Bevan M Northcote D Differential expression within a family of novel wound-induced genes in potato Mol Gen Genet 1989 215 200 208 10.1007/BF00339718 2710099

[B90] Ponstein AS Bres-Vloemans SA Sela-Buurlage MB van den Elzen PJ Melchers LS Cornelissen BJ A novel pathogen- and wound-inducible tobacco (*Nicotiana tabacum*) protein with antifungal activity Plant Physiol 1994 104 109 118 10.1104/pp.104.1.109 8115541 PMC159168

[B91] Liu F Yoo BC Lee JY Pan W Harmon AC Calcium-regulated phosphorylation of soybean serine acetyltransferase in response to oxidative stress J Biol Chem 2006 281 27405 27415 10.1074/jbc.M604548200 16854983

[B92] Arimura G Huber DP Bohlmann J Forest tent caterpillars (*Malacosoma disstria*) induce local and systemic diurnal emissions of terpenoid volatiles in hybrid poplar (*Populus trichocarpa *x *deltoides*): cDNA cloning, functional characterization, and patterns of gene expression of (-)-germacrene D synthase, PtdTPS1 Plant J 2004 37 603 616 10.1111/j.1365-313X.2003.01987.x 14756770

[B93] Molina A Segura A Garcia-Olmedo F Lipid transfer proteins (nsLTPs) from barley and maize leaves are potent inhibitors of bacterial and fungal plant pathogens FEBS Lett 1993 316 119 122 10.1016/0014-5793(93)81198-9 8420795

[B94] Ilmonen P Penn DJ Damjanovich K Morrison L Ghotbi L Potts WK Major histocompatibility complex heterozygosity reduces fitness in experimentally infected mice Genetics 2007 176 2501 2508 10.1534/genetics.107.074815 17603099 PMC1950649

[B95] Makino Y Okamoto K Yoshikawa N Aoshima M Hirota K Yodoi J Umesono K Makino I Tanaka H Thioredoxin: a redox-regulating cellular cofactor for glucocorticoid hormone action. Cross talk between endocrine control of stress response and cellular antioxidant defense system J Clin Invest 1996 98 2469 2477 10.1172/JCI119065 8958209 PMC507704

[B96] Takahashi H Ishihara T Hase S Chiba A Nakaho K Arie T Teraoka T Iwata M Tugane T Shibata D Beta-cyanoalanine synthase as a molecular marker for induced resistance by fungal glycoprotein elicitor and commercial plant activators Phytopathology 2006 96 908 916 10.1094/PHYTO-96-0908 18943757

[B97] Nembaware V Seoighe C Sayed M Gehring C A plant natriuretic peptide-like gene in the bacterial pathogen *Xanthomonas axonopodis *may induce hyper-hydration in the plant host: a hypothesis of molecular mimicry BMC Evol Biol 2004 4 10 10.1186/1471-2148-4-10 15038836 PMC387824

[B98] Poppenberger B Berthiller F Lucyshyn D Sieberer T Schuhmacher R Krska R Kuchler K Glossl J Luschnig C Adam G Detoxification of the *Fusarium *mycotoxin deoxynivalenol by a UDP-glucosyltransferase from *Arabidopsis thaliana* J Biol Chem 2003 278 47905 47914 10.1074/jbc.M307552200 12970342

[B99] Murray MG Thompson WF Rapid isolation of high molecular weight plant DNA Nucleic Acids Res 1980 8 4321 4325 10.1093/nar/8.19.4321 7433111 PMC324241

[B100] HuaDa Gene http://www.genomics.org.cn/

[B101] NCBI http://blast.ncbi.nlm.nih.gov/Blast.cgi

[B102] Morrissy AS Morin RD Delaney A Zeng T McDonald H Jones S Zhao Y Hirst M Marra MA Next-generation tag sequencing for cancer gene expression profiling Genome Res 2009 19 1825 1835 10.1101/gr.094482.109 19541910 PMC2765282

[B103] Audic S Claverie JM The significance of digital gene expression profiles Genome Res 1997 7 986 995 9331369 10.1101/gr.7.10.986

[B104] Benjamini Y Drai D Elmer G Kafkafi N Golani I Controlling the false discovery rate in behavior genetics research Behav Brain Res 2001 125 279 284 10.1016/S0166-4328(01)00297-2 11682119

[B105] Livak KJ Schmittgen TD Analysis of relative gene expression data using real-time quantitative PCR and the 2(-Delta Delta C(T)) Method Methods 2001 25 402 408 10.1006/meth.2001.1262 11846609

[B106] KEGG http://www.genome.jp/kegg/

[B107] Benjamini Y Hochberg Y Controlling the false discovery rate: a practical and powerful approach to multiple testing Journal of the Royal Statistical Society, Series B (Methodological) 1995 57 289 300

