# Supplementary material for: Whole genome wide expression profiles of Vitis amurensis grape responding to downy mildew by using Solexa sequencing technology
Source: BMC Plant Biol. 2010 Oct 28;10:234. doi: 10.1186/1471-2229-10-234 (PMC3017854; doi:10.1186/1471-2229-10-234)
Supplement: Additional file 2 — Complete list of involved pathways for upregualted DEGs. Pathways with Q value < 0.05 are significantly enriched for upregulated DEGs. [file 1471-2229-10-234-S2.DOC]

**Pathways enrichment for upregulated DEGs**

| # | Pathway | DEGs tested | Pvalue | Qvalue | Pathway ID |
| --- | --- | --- | --- | --- | --- |
| 1 | [Ribosome](../../../../C:%5CUsers%5Cwujiao%5CDesktop%5C20100324%5Cpathway%5CzsysmvszsyCK_Up.htm#gene1) | 53 (4.36%) | 0.0003533584 | 0.04063622 | ko03010 |
| 2 | [Amino sugar and nucleotide sugar metabolism](../../../../C:%5CUsers%5Cwujiao%5CDesktop%5C20100324%5Cpathway%5CzsysmvszsyCK_Up.htm#gene2) | 25 (2.06%) | 0.0009797429 | 0.05633522 | ko00520 |
| 3 | [Glycolysis / Gluconeogenesis](../../../../C:%5CUsers%5Cwujiao%5CDesktop%5C20100324%5Cpathway%5CzsysmvszsyCK_Up.htm#gene3) | 28 (2.3%) | 0.004330702 | 0.16601024 | ko00010 |
| 4 | [Biosynthesis of alkaloids derived from histidine and purine](../../../../C:%5CUsers%5Cwujiao%5CDesktop%5C20100324%5Cpathway%5CzsysmvszsyCK_Up.htm#gene4) | 31 (2.55%) | 0.01264678 | 0.36359493 | ko01065 |
| 5 | [Biosynthesis of alkaloids derived from ornithine, lysine and nicotinic acid](../../../../C:%5CUsers%5Cwujiao%5CDesktop%5C20100324%5Cpathway%5CzsysmvszsyCK_Up.htm#gene5) | 35 (2.88%) | 0.02071211 | 0.44594125 | ko01064 |
| 6 | [Starch and sucrose metabolism](../../../../C:%5CUsers%5Cwujiao%5CDesktop%5C20100324%5Cpathway%5CzsysmvszsyCK_Up.htm#gene6) | 49 (4.03%) | 0.0232665 | 0.44594125 | ko00500 |
| 7 | [Biosynthesis of alkaloids derived from shikimate pathway](../../../../C:%5CUsers%5Cwujiao%5CDesktop%5C20100324%5Cpathway%5CzsysmvszsyCK_Up.htm#gene7) | 39 (3.21%) | 0.03609649 | 0.58676105 | ko01063 |
| 8 | [N-Glycan biosynthesis](../../../../C:%5CUsers%5Cwujiao%5CDesktop%5C20100324%5Cpathway%5CzsysmvszsyCK_Up.htm#gene8) | 10 (0.82%) | 0.05281267 | 0.58676105 | ko00510 |
| 9 | [Fructose and mannose metabolism](../../../../C:%5CUsers%5Cwujiao%5CDesktop%5C20100324%5Cpathway%5CzsysmvszsyCK_Up.htm#gene9) | 14 (1.15%) | 0.05598061 | 0.58676105 | ko00051 |
| 10 | [Selenoamino acid metabolism](../../../../C:%5CUsers%5Cwujiao%5CDesktop%5C20100324%5Cpathway%5CzsysmvszsyCK_Up.htm#gene10) | 11 (0.91%) | 0.058659 | 0.58676105 | ko00450 |
| 11 | [Endocytosis](../../../../C:%5CUsers%5Cwujiao%5CDesktop%5C20100324%5Cpathway%5CzsysmvszsyCK_Up.htm#gene11) | 19 (1.56%) | 0.0611623 | 0.58676105 | ko04144 |
| 12 | [Glyoxylate and dicarboxylate metabolism](../../../../C:%5CUsers%5Cwujiao%5CDesktop%5C20100324%5Cpathway%5CzsysmvszsyCK_Up.htm#gene12) | 9 (0.74%) | 0.06465981 | 0.58676105 | ko00630 |
| 13 | [Biosynthesis of plant hormones](../../../../C:%5CUsers%5Cwujiao%5CDesktop%5C20100324%5Cpathway%5CzsysmvszsyCK_Up.htm#gene13) | 82 (6.75%) | 0.06632951 | 0.58676105 | ko01070 |
| 14 | [Glycerolipid metabolism](../../../../C:%5CUsers%5Cwujiao%5CDesktop%5C20100324%5Cpathway%5CzsysmvszsyCK_Up.htm#gene14) | 14 (1.15%) | 0.07520365 | 0.61774427 | ko00561 |
| 15 | [Pentose phosphate pathway](../../../../C:%5CUsers%5Cwujiao%5CDesktop%5C20100324%5Cpathway%5CzsysmvszsyCK_Up.htm#gene15) | 9 (0.74%) | 0.08156704 | 0.62534731 | ko00030 |
| 16 | [Glucosinolate biosynthesis](../../../../C:%5CUsers%5Cwujiao%5CDesktop%5C20100324%5Cpathway%5CzsysmvszsyCK_Up.htm#gene16) | 13 (1.07%) | 0.1062271 | 0.74686141 | ko00966 |
| 17 | [Linoleic acid metabolism](../../../../C:%5CUsers%5Cwujiao%5CDesktop%5C20100324%5Cpathway%5CzsysmvszsyCK_Up.htm#gene17) | 15 (1.23%) | 0.1104056 | 0.74686141 | ko00591 |
| 18 | [SNARE interactions in vesicular transport](../../../../C:%5CUsers%5Cwujiao%5CDesktop%5C20100324%5Cpathway%5CzsysmvszsyCK_Up.htm#gene18) | 8 (0.66%) | 0.1324604 | 0.82576018 | ko04130 |
| 19 | [Glycerophospholipid metabolism](../../../../C:%5CUsers%5Cwujiao%5CDesktop%5C20100324%5Cpathway%5CzsysmvszsyCK_Up.htm#gene19) | 13 (1.07%) | 0.1625328 | 0.82576018 | ko00564 |
| 20 | [Thiamine metabolism](../../../../C:%5CUsers%5Cwujiao%5CDesktop%5C20100324%5Cpathway%5CzsysmvszsyCK_Up.htm#gene20) | 3 (0.25%) | 0.1671543 | 0.82576018 | ko00730 |
| 21 | [Galactose metabolism](../../../../C:%5CUsers%5Cwujiao%5CDesktop%5C20100324%5Cpathway%5CzsysmvszsyCK_Up.htm#gene21) | 11 (0.91%) | 0.168421 | 0.82576018 | ko00052 |
| 22 | [Carbon fixation in photosynthetic organisms](../../../../C:%5CUsers%5Cwujiao%5CDesktop%5C20100324%5Cpathway%5CzsysmvszsyCK_Up.htm#gene22) | 12 (0.99%) | 0.1693123 | 0.82576018 | ko00710 |
| 23 | [Arginine and proline metabolism](../../../../C:%5CUsers%5Cwujiao%5CDesktop%5C20100324%5Cpathway%5CzsysmvszsyCK_Up.htm#gene23) | 11 (0.91%) | 0.1763977 | 0.82576018 | ko00330 |
| 24 | [Glutathione metabolism](../../../../C:%5CUsers%5Cwujiao%5CDesktop%5C20100324%5Cpathway%5CzsysmvszsyCK_Up.htm#gene24) | 16 (1.32%) | 0.1873108 | 0.82576018 | ko00480 |
| 25 | [Phenylpropanoid biosynthesis](../../../../C:%5CUsers%5Cwujiao%5CDesktop%5C20100324%5Cpathway%5CzsysmvszsyCK_Up.htm#gene25) | 58 (4.77%) | 0.1883479 | 0.82576018 | ko00940 |
| 26 | [Ubiquitin mediated proteolysis](../../../../C:%5CUsers%5Cwujiao%5CDesktop%5C20100324%5Cpathway%5CzsysmvszsyCK_Up.htm#gene26) | 28 (2.3%) | 0.1894998 | 0.82576018 | ko04120 |
| 27 | [Carotenoid biosynthesis](../../../../C:%5CUsers%5Cwujiao%5CDesktop%5C20100324%5Cpathway%5CzsysmvszsyCK_Up.htm#gene27) | 14 (1.15%) | 0.1977340 | 0.82576018 | ko00906 |
| 28 | [Sulfur metabolism](../../../../C:%5CUsers%5Cwujiao%5CDesktop%5C20100324%5Cpathway%5CzsysmvszsyCK_Up.htm#gene28) | 6 (0.49%) | 0.2025984 | 0.82576018 | ko00920 |
| 29 | [Biosynthesis of alkaloids derived from terpenoid and polyketide](../../../../C:%5CUsers%5Cwujiao%5CDesktop%5C20100324%5Cpathway%5CzsysmvszsyCK_Up.htm#gene29) | 28 (2.3%) | 0.2144095 | 0.82576018 | ko01066 |
| 30 | [Regulation of autophagy](../../../../C:%5CUsers%5Cwujiao%5CDesktop%5C20100324%5Cpathway%5CzsysmvszsyCK_Up.htm#gene30) | 6 (0.49%) | 0.2154157 | 0.82576018 | ko04140 |
| 31 | [Anthocyanin biosynthesis](../../../../C:%5CUsers%5Cwujiao%5CDesktop%5C20100324%5Cpathway%5CzsysmvszsyCK_Up.htm#gene31) | 7 (0.58%) | 0.2430823 | 0.86912609 | ko00942 |
| 32 | [Spliceosome](../../../../C:%5CUsers%5Cwujiao%5CDesktop%5C20100324%5Cpathway%5CzsysmvszsyCK_Up.htm#gene32) | 48 (3.95%) | 0.2464756 | 0.86912609 | ko03040 |
| 33 | [Metabolism of xenobiotics by cytochrome P450](../../../../C:%5CUsers%5Cwujiao%5CDesktop%5C20100324%5Cpathway%5CzsysmvszsyCK_Up.htm#gene33) | 18 (1.48%) | 0.2494014 | 0.86912609 | ko00980 |
| 34 | [Glycine, serine and threonine metabolism](../../../../C:%5CUsers%5Cwujiao%5CDesktop%5C20100324%5Cpathway%5CzsysmvszsyCK_Up.htm#gene34) | 9 (0.74%) | 0.2951433 | 0.99827881 | ko00260 |
| 35 | [Cyanoamino acid metabolism](../../../../C:%5CUsers%5Cwujiao%5CDesktop%5C20100324%5Cpathway%5CzsysmvszsyCK_Up.htm#gene35) | 23 (1.89%) | 0.3232934 | 0.99999820 | ko00460 |
| 36 | [Citrate cycle (TCA cycle)](../../../../C:%5CUsers%5Cwujiao%5CDesktop%5C20100324%5Cpathway%5CzsysmvszsyCK_Up.htm#gene36) | 9 (0.74%) | 0.3287696 | 0.99999820 | ko00020 |
| 37 | [Pentose and glucuronate interconversions](../../../../C:%5CUsers%5Cwujiao%5CDesktop%5C20100324%5Cpathway%5CzsysmvszsyCK_Up.htm#gene37) | 16 (1.32%) | 0.3302179 | 0.99999820 | ko00040 |
| 38 | [Ether lipid metabolism](../../../../C:%5CUsers%5Cwujiao%5CDesktop%5C20100324%5Cpathway%5CzsysmvszsyCK_Up.htm#gene38) | 5 (0.41%) | 0.3642860 | 0.99999820 | ko00565 |
| 39 | [Flavonoid biosynthesis](../../../../C:%5CUsers%5Cwujiao%5CDesktop%5C20100324%5Cpathway%5CzsysmvszsyCK_Up.htm#gene39) | 45 (3.7%) | 0.3668869 | 0.99999820 | ko00941 |
| 40 | [Sphingolipid metabolism](../../../../C:%5CUsers%5Cwujiao%5CDesktop%5C20100324%5Cpathway%5CzsysmvszsyCK_Up.htm#gene40) | 6 (0.49%) | 0.3690742 | 0.99999820 | ko00600 |
| 41 | [alpha-Linolenic acid metabolism](../../../../C:%5CUsers%5Cwujiao%5CDesktop%5C20100324%5Cpathway%5CzsysmvszsyCK_Up.htm#gene41) | 16 (1.32%) | 0.3718613 | 0.99999820 | ko00592 |
| 42 | [Phosphatidylinositol signaling system](../../../../C:%5CUsers%5Cwujiao%5CDesktop%5C20100324%5Cpathway%5CzsysmvszsyCK_Up.htm#gene42) | 12 (0.99%) | 0.3746693 | 0.99999820 | ko04070 |
| 43 | [Biosynthesis of phenylpropanoids](../../../../C:%5CUsers%5Cwujiao%5CDesktop%5C20100324%5Cpathway%5CzsysmvszsyCK_Up.htm#gene43) | 80 (6.58%) | 0.3761452 | 0.99999820 | ko01061 |
| 44 | [Lipoic acid metabolism](../../../../C:%5CUsers%5Cwujiao%5CDesktop%5C20100324%5Cpathway%5CzsysmvszsyCK_Up.htm#gene44) | 1 (0.08%) | 0.3910785 | 0.99999820 | ko00785 |
| 45 | [Nicotinate and nicotinamide metabolism](../../../../C:%5CUsers%5Cwujiao%5CDesktop%5C20100324%5Cpathway%5CzsysmvszsyCK_Up.htm#gene45) | 2 (0.16%) | 0.4239257 | 0.99999820 | ko00760 |
| 46 | [Tryptophan metabolism](../../../../C:%5CUsers%5Cwujiao%5CDesktop%5C20100324%5Cpathway%5CzsysmvszsyCK_Up.htm#gene46) | 18 (1.48%) | 0.4337001 | 0.99999820 | ko00380 |
| 47 | [Tyrosine metabolism](../../../../C:%5CUsers%5Cwujiao%5CDesktop%5C20100324%5Cpathway%5CzsysmvszsyCK_Up.htm#gene47) | 11 (0.91%) | 0.4371867 | 0.99999820 | ko00350 |
| 48 | [Taurine and hypotaurine metabolism](../../../../C:%5CUsers%5Cwujiao%5CDesktop%5C20100324%5Cpathway%5CzsysmvszsyCK_Up.htm#gene48) | 1 (0.08%) | 0.4394087 | 0.99999820 | ko00430 |
| 49 | [Fatty acid elongation in mitochondria](../../../../C:%5CUsers%5Cwujiao%5CDesktop%5C20100324%5Cpathway%5CzsysmvszsyCK_Up.htm#gene49) | 1 (0.08%) | 0.4394087 | 0.99999820 | ko00062 |
| 50 | [Biotin metabolism](../../../../C:%5CUsers%5Cwujiao%5CDesktop%5C20100324%5Cpathway%5CzsysmvszsyCK_Up.htm#gene50) | 1 (0.08%) | 0.4394087 | 0.99999820 | ko00780 |
| 51 | [beta-Alanine metabolism](../../../../C:%5CUsers%5Cwujiao%5CDesktop%5C20100324%5Cpathway%5CzsysmvszsyCK_Up.htm#gene51) | 6 (0.49%) | 0.4559292 | 0.99999820 | ko00410 |
| 52 | [Phenylalanine, tyrosine and tryptophan biosynthesis](../../../../C:%5CUsers%5Cwujiao%5CDesktop%5C20100324%5Cpathway%5CzsysmvszsyCK_Up.htm#gene52) | 6 (0.49%) | 0.4701938 | 0.99999820 | ko00400 |
| 53 | [Biosynthesis of terpenoids and steroids](../../../../C:%5CUsers%5Cwujiao%5CDesktop%5C20100324%5Cpathway%5CzsysmvszsyCK_Up.htm#gene53) | 49 (4.03%) | 0.4712886 | 0.99999820 | ko01062 |
| 54 | [Pyruvate metabolism](../../../../C:%5CUsers%5Cwujiao%5CDesktop%5C20100324%5Cpathway%5CzsysmvszsyCK_Up.htm#gene54) | 11 (0.91%) | 0.4989662 | 0.99999820 | ko00620 |
| 55 | [Pantothenate and CoA biosynthesis](../../../../C:%5CUsers%5Cwujiao%5CDesktop%5C20100324%5Cpathway%5CzsysmvszsyCK_Up.htm#gene55) | 4 (0.33%) | 0.5011226 | 0.99999820 | ko00770 |
| 56 | [RNA degradation](../../../../C:%5CUsers%5Cwujiao%5CDesktop%5C20100324%5Cpathway%5CzsysmvszsyCK_Up.htm#gene56) | 13 (1.07%) | 0.5250507 | 0.99999820 | ko03018 |
| 57 | [Isoquinoline alkaloid biosynthesis](../../../../C:%5CUsers%5Cwujiao%5CDesktop%5C20100324%5Cpathway%5CzsysmvszsyCK_Up.htm#gene57) | 4 (0.33%) | 0.5352912 | 0.99999820 | ko00950 |
| 58 | [Basal transcription factors](../../../../C:%5CUsers%5Cwujiao%5CDesktop%5C20100324%5Cpathway%5CzsysmvszsyCK_Up.htm#gene58) | 7 (0.58%) | 0.5663183 | 0.99999820 | ko03022 |
| 59 | [Riboflavin metabolism](../../../../C:%5CUsers%5Cwujiao%5CDesktop%5C20100324%5Cpathway%5CzsysmvszsyCK_Up.htm#gene59) | 2 (0.16%) | 0.5782229 | 0.99999820 | ko00740 |
| 60 | [Butanoate metabolism](../../../../C:%5CUsers%5Cwujiao%5CDesktop%5C20100324%5Cpathway%5CzsysmvszsyCK_Up.htm#gene60) | 11 (0.91%) | 0.6066106 | 0.99999820 | ko00650 |
| 61 | [Brassinosteroid biosynthesis](../../../../C:%5CUsers%5Cwujiao%5CDesktop%5C20100324%5Cpathway%5CzsysmvszsyCK_Up.htm#gene61) | 3 (0.25%) | 0.6077089 | 0.99999820 | ko00905 |
| 62 | [Natural killer cell mediated cytotoxicity](../../../../C:%5CUsers%5Cwujiao%5CDesktop%5C20100324%5Cpathway%5CzsysmvszsyCK_Up.htm#gene62) | 5 (0.41%) | 0.6224466 | 0.99999820 | ko04650 |
| 63 | [Vitamin B6 metabolism](../../../../C:%5CUsers%5Cwujiao%5CDesktop%5C20100324%5Cpathway%5CzsysmvszsyCK_Up.htm#gene63) | 1 (0.08%) | 0.6292897 | 0.99999820 | ko00750 |
| 64 | [Lysine biosynthesis](../../../../C:%5CUsers%5Cwujiao%5CDesktop%5C20100324%5Cpathway%5CzsysmvszsyCK_Up.htm#gene64) | 2 (0.16%) | 0.6432084 | 0.99999820 | ko00300 |
| 65 | [Polyketide sugar unit biosynthesis](../../../../C:%5CUsers%5Cwujiao%5CDesktop%5C20100324%5Cpathway%5CzsysmvszsyCK_Up.htm#gene65) | 1 (0.08%) | 0.6587246 | 0.99999820 | ko00523 |
| 66 | [Glycosphingolipid biosynthesis - globo series](../../../../C:%5CUsers%5Cwujiao%5CDesktop%5C20100324%5Cpathway%5CzsysmvszsyCK_Up.htm#gene66) | 1 (0.08%) | 0.6858241 | 0.99999820 | ko00603 |
| 67 | [Tropane, piperidine and pyridine alkaloid biosynthesis](../../../../C:%5CUsers%5Cwujiao%5CDesktop%5C20100324%5Cpathway%5CzsysmvszsyCK_Up.htm#gene67) | 4 (0.33%) | 0.6858523 | 0.99999820 | ko00960 |
| 68 | [Alanine, aspartate and glutamate metabolism](../../../../C:%5CUsers%5Cwujiao%5CDesktop%5C20100324%5Cpathway%5CzsysmvszsyCK_Up.htm#gene68) | 9 (0.74%) | 0.6861948 | 0.99999820 | ko00250 |
| 69 | [Phenylalanine metabolism](../../../../C:%5CUsers%5Cwujiao%5CDesktop%5C20100324%5Cpathway%5CzsysmvszsyCK_Up.htm#gene69) | 12 (0.99%) | 0.7053478 | 0.99999820 | ko00360 |
| 70 | [Benzoxazinoid biosynthesis](../../../../C:%5CUsers%5Cwujiao%5CDesktop%5C20100324%5Cpathway%5CzsysmvszsyCK_Up.htm#gene70) | 9 (0.74%) | 0.7210209 | 0.99999820 | ko00402 |
| 71 | [Fatty acid biosynthesis](../../../../C:%5CUsers%5Cwujiao%5CDesktop%5C20100324%5Cpathway%5CzsysmvszsyCK_Up.htm#gene71) | 5 (0.41%) | 0.7411899 | 0.99999820 | ko00061 |
| 72 | [Ubiquinone and other terpenoid-quinone biosynthesis](../../../../C:%5CUsers%5Cwujiao%5CDesktop%5C20100324%5Cpathway%5CzsysmvszsyCK_Up.htm#gene72) | 5 (0.41%) | 0.7513931 | 0.99999820 | ko00130 |
| 73 | [Zeatin biosynthesis](../../../../C:%5CUsers%5Cwujiao%5CDesktop%5C20100324%5Cpathway%5CzsysmvszsyCK_Up.htm#gene73) | 5 (0.41%) | 0.7612938 | 0.99999820 | ko00908 |
| 74 | [Methane metabolism](../../../../C:%5CUsers%5Cwujiao%5CDesktop%5C20100324%5Cpathway%5CzsysmvszsyCK_Up.htm#gene74) | 10 (0.82%) | 0.7679758 | 0.99999820 | ko00680 |
| 75 | [Synthesis and degradation of ketone bodies](../../../../C:%5CUsers%5Cwujiao%5CDesktop%5C20100324%5Cpathway%5CzsysmvszsyCK_Up.htm#gene75) | 1 (0.08%) | 0.7922814 | 0.99999820 | ko00072 |
| 76 | [Protein export](../../../../C:%5CUsers%5Cwujiao%5CDesktop%5C20100324%5Cpathway%5CzsysmvszsyCK_Up.htm#gene76) | 1 (0.08%) | 0.808782 | 0.99999820 | ko03060 |
| 77 | [Glycosaminoglycan degradation](../../../../C:%5CUsers%5Cwujiao%5CDesktop%5C20100324%5Cpathway%5CzsysmvszsyCK_Up.htm#gene77) | 2 (0.16%) | 0.826799 | 0.99999820 | ko00531 |
| 78 | [Indole alkaloid biosynthesis](../../../../C:%5CUsers%5Cwujiao%5CDesktop%5C20100324%5Cpathway%5CzsysmvszsyCK_Up.htm#gene78) | 4 (0.33%) | 0.8332405 | 0.99999820 | ko00901 |
| 79 | [Peroxisome](../../../../C:%5CUsers%5Cwujiao%5CDesktop%5C20100324%5Cpathway%5CzsysmvszsyCK_Up.htm#gene79) | 14 (1.15%) | 0.8446981 | 0.99999820 | ko04146 |
| 80 | [Porphyrin and chlorophyll metabolism](../../../../C:%5CUsers%5Cwujiao%5CDesktop%5C20100324%5Cpathway%5CzsysmvszsyCK_Up.htm#gene80) | 4 (0.33%) | 0.8486837 | 0.99999820 | ko00860 |
| 81 | [Arachidonic acid metabolism](../../../../C:%5CUsers%5Cwujiao%5CDesktop%5C20100324%5Cpathway%5CzsysmvszsyCK_Up.htm#gene81) | 1 (0.08%) | 0.8508328 | 0.99999820 | ko00590 |
| 82 | [Biosynthesis of unsaturated fatty acids](../../../../C:%5CUsers%5Cwujiao%5CDesktop%5C20100324%5Cpathway%5CzsysmvszsyCK_Up.htm#gene82) | 8 (0.66%) | 0.8652043 | 0.99999820 | ko01040 |
| 83 | [Cysteine and methionine metabolism](../../../../C:%5CUsers%5Cwujiao%5CDesktop%5C20100324%5Cpathway%5CzsysmvszsyCK_Up.htm#gene83) | 18 (1.48%) | 0.8800483 | 0.99999820 | ko00270 |
| 84 | [Proteasome](../../../../C:%5CUsers%5Cwujiao%5CDesktop%5C20100324%5Cpathway%5CzsysmvszsyCK_Up.htm#gene84) | 4 (0.33%) | 0.898869 | 0.99999820 | ko03050 |
| 85 | [Terpenoid backbone biosynthesis](../../../../C:%5CUsers%5Cwujiao%5CDesktop%5C20100324%5Cpathway%5CzsysmvszsyCK_Up.htm#gene85) | 6 (0.49%) | 0.9104849 | 0.99999820 | ko00900 |
| 86 | [Non-homologous end-joining](../../../../C:%5CUsers%5Cwujiao%5CDesktop%5C20100324%5Cpathway%5CzsysmvszsyCK_Up.htm#gene86) | 1 (0.08%) | 0.916454 | 0.99999820 | ko03450 |
| 87 | [Fatty acid metabolism](../../../../C:%5CUsers%5Cwujiao%5CDesktop%5C20100324%5Cpathway%5CzsysmvszsyCK_Up.htm#gene87) | 8 (0.66%) | 0.9289326 | 0.99999820 | ko00071 |
| 88 | [Glycosphingolipid biosynthesis - ganglio series](../../../../C:%5CUsers%5Cwujiao%5CDesktop%5C20100324%5Cpathway%5CzsysmvszsyCK_Up.htm#gene88) | 1 (0.08%) | 0.9292094 | 0.99999820 | ko00604 |
| 89 | [Circadian rhythm - plant](../../../../C:%5CUsers%5Cwujiao%5CDesktop%5C20100324%5Cpathway%5CzsysmvszsyCK_Up.htm#gene89) | 14 (1.15%) | 0.9296028 | 0.99999820 | ko04712 |
| 90 | [Lysine degradation](../../../../C:%5CUsers%5Cwujiao%5CDesktop%5C20100324%5Cpathway%5CzsysmvszsyCK_Up.htm#gene90) | 4 (0.33%) | 0.9337707 | 0.99999820 | ko00310 |
| 91 | [Propanoate metabolism](../../../../C:%5CUsers%5Cwujiao%5CDesktop%5C20100324%5Cpathway%5CzsysmvszsyCK_Up.htm#gene91) | 4 (0.33%) | 0.9405905 | 0.99999820 | ko00640 |
| 92 | [Base excision repair](../../../../C:%5CUsers%5Cwujiao%5CDesktop%5C20100324%5Cpathway%5CzsysmvszsyCK_Up.htm#gene92) | 5 (0.41%) | 0.9486342 | 0.99999820 | ko03410 |
| 93 | [Inositol phosphate metabolism](../../../../C:%5CUsers%5Cwujiao%5CDesktop%5C20100324%5Cpathway%5CzsysmvszsyCK_Up.htm#gene93) | 3 (0.25%) | 0.9498757 | 0.99999820 | ko00562 |
| 94 | [Stilbenoid, diarylheptanoid and gingerol biosynthesis](../../../../C:%5CUsers%5Cwujiao%5CDesktop%5C20100324%5Cpathway%5CzsysmvszsyCK_Up.htm#gene94) | 24 (1.98%) | 0.9524244 | 0.99999820 | ko00945 |
| 95 | [Diterpenoid biosynthesis](../../../../C:%5CUsers%5Cwujiao%5CDesktop%5C20100324%5Cpathway%5CzsysmvszsyCK_Up.htm#gene95) | 7 (0.58%) | 0.9544997 | 0.99999820 | ko00904 |
| 96 | [Valine, leucine and isoleucine degradation](../../../../C:%5CUsers%5Cwujiao%5CDesktop%5C20100324%5Cpathway%5CzsysmvszsyCK_Up.htm#gene96) | 4 (0.33%) | 0.9549298 | 0.99999820 | ko00280 |
| 97 | [One carbon pool by folate](../../../../C:%5CUsers%5Cwujiao%5CDesktop%5C20100324%5Cpathway%5CzsysmvszsyCK_Up.htm#gene97) | 1 (0.08%) | 0.9569408 | 0.99999820 | ko00670 |
| 98 | [DNA replication](../../../../C:%5CUsers%5Cwujiao%5CDesktop%5C20100324%5Cpathway%5CzsysmvszsyCK_Up.htm#gene98) | 5 (0.41%) | 0.9582548 | 0.99999820 | ko03030 |
| 99 | [Other glycan degradation](../../../../C:%5CUsers%5Cwujiao%5CDesktop%5C20100324%5Cpathway%5CzsysmvszsyCK_Up.htm#gene99) | 2 (0.16%) | 0.9625526 | 0.99999820 | ko00511 |
| 100 | [Limonene and pinene degradation](../../../../C:%5CUsers%5Cwujiao%5CDesktop%5C20100324%5Cpathway%5CzsysmvszsyCK_Up.htm#gene100) | 18 (1.48%) | 0.9664413 | 0.99999820 | ko00903 |
| 101 | [Purine metabolism](../../../../C:%5CUsers%5Cwujiao%5CDesktop%5C20100324%5Cpathway%5CzsysmvszsyCK_Up.htm#gene101) | 12 (0.99%) | 0.9814211 | 0.99999820 | ko00230 |
| 102 | [Valine, leucine and isoleucine biosynthesis](../../../../C:%5CUsers%5Cwujiao%5CDesktop%5C20100324%5Cpathway%5CzsysmvszsyCK_Up.htm#gene102) | 2 (0.16%) | 0.981447 | 0.99999820 | ko00290 |
| 103 | [Nitrogen metabolism](../../../../C:%5CUsers%5Cwujiao%5CDesktop%5C20100324%5Cpathway%5CzsysmvszsyCK_Up.htm#gene103) | 9 (0.74%) | 0.9833426 | 0.99999820 | ko00910 |
| 104 | [Nucleotide excision repair](../../../../C:%5CUsers%5Cwujiao%5CDesktop%5C20100324%5Cpathway%5CzsysmvszsyCK_Up.htm#gene104) | 5 (0.41%) | 0.9859516 | 0.99999820 | ko03420 |
| 105 | [Aminoacyl-tRNA biosynthesis](../../../../C:%5CUsers%5Cwujiao%5CDesktop%5C20100324%5Cpathway%5CzsysmvszsyCK_Up.htm#gene105) | 5 (0.41%) | 0.9867233 | 0.99999820 | ko00970 |
| 106 | [Mismatch repair](../../../../C:%5CUsers%5Cwujiao%5CDesktop%5C20100324%5Cpathway%5CzsysmvszsyCK_Up.htm#gene106) | 2 (0.16%) | 0.989537 | 0.99999820 | ko03430 |
| 107 | [Metabolic pathways](../../../../C:%5CUsers%5Cwujiao%5CDesktop%5C20100324%5Cpathway%5CzsysmvszsyCK_Up.htm#gene107) | 274 (22.55%) | 0.9900928 | 0.99999820 | ko01100 |
| 108 | [Ascorbate and aldarate metabolism](../../../../C:%5CUsers%5Cwujiao%5CDesktop%5C20100324%5Cpathway%5CzsysmvszsyCK_Up.htm#gene108) | 7 (0.58%) | 0.9911425 | 0.99999820 | ko00053 |
| 109 | [Flavone and flavonol biosynthesis](../../../../C:%5CUsers%5Cwujiao%5CDesktop%5C20100324%5Cpathway%5CzsysmvszsyCK_Up.htm#gene109) | 9 (0.74%) | 0.9918617 | 0.99999820 | ko00944 |
| 110 | [Pyrimidine metabolism](../../../../C:%5CUsers%5Cwujiao%5CDesktop%5C20100324%5Cpathway%5CzsysmvszsyCK_Up.htm#gene110) | 10 (0.82%) | 0.9962046 | 0.99999820 | ko00240 |
| 111 | [Steroid biosynthesis](../../../../C:%5CUsers%5Cwujiao%5CDesktop%5C20100324%5Cpathway%5CzsysmvszsyCK_Up.htm#gene111) | 2 (0.16%) | 0.9993225 | 0.99999820 | ko00100 |
| 112 | [Oxidative phosphorylation](../../../../C:%5CUsers%5Cwujiao%5CDesktop%5C20100324%5Cpathway%5CzsysmvszsyCK_Up.htm#gene112) | 12 (0.99%) | 0.9994185 | 0.99999820 | ko00190 |
| 113 | [Monoterpenoid biosynthesis](../../../../C:%5CUsers%5Cwujiao%5CDesktop%5C20100324%5Cpathway%5CzsysmvszsyCK_Up.htm#gene113) | 2 (0.16%) | 0.9995698 | 0.99999820 | ko00902 |
| 114 | [ABC transporters](../../../../C:%5CUsers%5Cwujiao%5CDesktop%5C20100324%5Cpathway%5CzsysmvszsyCK_Up.htm#gene114) | 5 (0.41%) | 0.9998705 | 0.99999820 | ko02010 |
| 115 | [Photosynthesis](../../../../C:%5CUsers%5Cwujiao%5CDesktop%5C20100324%5Cpathway%5CzsysmvszsyCK_Up.htm#gene115) | 1 (0.08%) | 0.9999982 | 0.99999820 | ko00195 |
