# Supplementary material for: Whole genome wide expression profiles of Vitis amurensis grape responding to downy mildew by using Solexa sequencing technology
Source: BMC Plant Biol. 2010 Oct 28;10:234. doi: 10.1186/1471-2229-10-234 (PMC3017854; doi:10.1186/1471-2229-10-234)
Supplement: Additional file 3 — Complete list of involved pathways for downregualted DEGs. Pathways with Q value < 0.05 are significantly enriched for downregulated DEGs. [file 1471-2229-10-234-S3.DOC]

**Pathway enrichment for downregulated DEGs**

| # | Pathway | DEGs tested | Pvalue | Qvalue | Pathway ID |
| --- | --- | --- | --- | --- | --- |
| 1 | [Photosynthesis](../../../../C:%5CUsers%5Cwujiao%5CDesktop%5C20100324%5Cpathway%5CzsysmvszsyCK_Down.htm#gene1) | 20 (3.14%) | 9.961282e-06 | 0.001065857 | ko00195 |
| 2 | [Photosynthesis - antenna proteins](../../../../C:%5CUsers%5Cwujiao%5CDesktop%5C20100324%5Cpathway%5CzsysmvszsyCK_Down.htm#gene2) | 6 (0.94%) | 4.225173e-05 | 0.002260468 | ko00196 |
| 3 | [Folate biosynthesis](../../../../C:%5CUsers%5Cwujiao%5CDesktop%5C20100324%5Cpathway%5CzsysmvszsyCK_Down.htm#gene3) | 5 (0.78%) | 0.0001794234 | 0.006399435 | ko00790 |
| 4 | [Nicotinate and nicotinamide metabolism](../../../../C:%5CUsers%5Cwujiao%5CDesktop%5C20100324%5Cpathway%5CzsysmvszsyCK_Down.htm#gene4) | 5 (0.78%) | 0.0006685412 | 0.012489297 | ko00760 |
| 5 | [Fructose and mannose metabolism](../../../../C:%5CUsers%5Cwujiao%5CDesktop%5C20100324%5Cpathway%5CzsysmvszsyCK_Down.htm#gene5) | 13 (2.04%) | 0.0007003344 | 0.012489297 | ko00051 |
| 6 | [Carbon fixation in photosynthetic organisms](../../../../C:%5CUsers%5Cwujiao%5CDesktop%5C20100324%5Cpathway%5CzsysmvszsyCK_Down.htm#gene6) | 13 (2.04%) | 0.0007003344 | 0.012489297 | ko00710 |
| 7 | [Pyruvate metabolism](../../../../C:%5CUsers%5Cwujiao%5CDesktop%5C20100324%5Cpathway%5CzsysmvszsyCK_Down.htm#gene7) | 14 (2.2%) | 0.001380648 | 0.020996704 | ko00620 |
| 8 | [Polyketide sugar unit biosynthesis](../../../../C:%5CUsers%5Cwujiao%5CDesktop%5C20100324%5Cpathway%5CzsysmvszsyCK_Down.htm#gene8) | 4 (0.63%) | 0.001569847 | 0.020996704 | ko00523 |
| 9 | [Purine metabolism](../../../../C:%5CUsers%5Cwujiao%5CDesktop%5C20100324%5Cpathway%5CzsysmvszsyCK_Down.htm#gene9) | 21 (3.3%) | 0.001809214 | 0.021509544 | ko00230 |
| 10 | [Biosynthesis of alkaloids derived from histidine and purine](../../../../C:%5CUsers%5Cwujiao%5CDesktop%5C20100324%5Cpathway%5CzsysmvszsyCK_Down.htm#gene10) | 21 (3.3%) | 0.002527123 | 0.027040216 | ko01065 |
| 11 | [Pentose phosphate pathway](../../../../C:%5CUsers%5Cwujiao%5CDesktop%5C20100324%5Cpathway%5CzsysmvszsyCK_Down.htm#gene11) | 8 (1.26%) | 0.00643259 | 0.058005895 | ko00030 |
| 12 | [Glycolysis / Gluconeogenesis](../../../../C:%5CUsers%5Cwujiao%5CDesktop%5C20100324%5Cpathway%5CzsysmvszsyCK_Down.htm#gene12) | 17 (2.67%) | 0.006505334 | 0.058005895 | ko00010 |
| 13 | [Circadian rhythm - plant](../../../../C:%5CUsers%5Cwujiao%5CDesktop%5C20100324%5Cpathway%5CzsysmvszsyCK_Down.htm#gene13) | 18 (2.83%) | 0.01467605 | 0.120795181 | ko04712 |
| 14 | [Pyrimidine metabolism](../../../../C:%5CUsers%5Cwujiao%5CDesktop%5C20100324%5Cpathway%5CzsysmvszsyCK_Down.htm#gene14) | 18 (2.83%) | 0.01766656 | 0.135022994 | ko00240 |
| 15 | [Glycosaminoglycan degradation](../../../../C:%5CUsers%5Cwujiao%5CDesktop%5C20100324%5Cpathway%5CzsysmvszsyCK_Down.htm#gene15) | 5 (0.78%) | 0.02193242 | 0.156451263 | ko00531 |
| 16 | [Linoleic acid metabolism](../../../../C:%5CUsers%5Cwujiao%5CDesktop%5C20100324%5Cpathway%5CzsysmvszsyCK_Down.htm#gene16) | 11 (1.73%) | 0.02408730 | 0.161083819 | ko00591 |
| 17 | [Glycine, serine and threonine metabolism](../../../../C:%5CUsers%5Cwujiao%5CDesktop%5C20100324%5Cpathway%5CzsysmvszsyCK_Down.htm#gene17) | 8 (1.26%) | 0.03551871 | 0.212098075 | ko00260 |
| 18 | [Metabolic pathways](../../../../C:%5CUsers%5Cwujiao%5CDesktop%5C20100324%5Cpathway%5CzsysmvszsyCK_Down.htm#gene18) | 181 (28.41%) | 0.03568005 | 0.212098075 | ko01100 |
| 19 | [Glycosphingolipid biosynthesis - ganglio series](../../../../C:%5CUsers%5Cwujiao%5CDesktop%5C20100324%5Cpathway%5CzsysmvszsyCK_Down.htm#gene19) | 4 (0.63%) | 0.04239424 | 0.238746509 | ko00604 |
| 20 | [Riboflavin metabolism](../../../../C:%5CUsers%5Cwujiao%5CDesktop%5C20100324%5Cpathway%5CzsysmvszsyCK_Down.htm#gene20) | 3 (0.47%) | 0.07586645 | 0.405885508 | ko00740 |
| 21 | [RNA polymerase](../../../../C:%5CUsers%5Cwujiao%5CDesktop%5C20100324%5Cpathway%5CzsysmvszsyCK_Down.htm#gene21) | 7 (1.1%) | 0.08452502 | 0.430675102 | ko03020 |
| 22 | [Biosynthesis of unsaturated fatty acids](../../../../C:%5CUsers%5Cwujiao%5CDesktop%5C20100324%5Cpathway%5CzsysmvszsyCK_Down.htm#gene22) | 9 (1.41%) | 0.1216181 | 0.591506214 | ko01040 |
| 23 | [Butanoate metabolism](../../../../C:%5CUsers%5Cwujiao%5CDesktop%5C20100324%5Cpathway%5CzsysmvszsyCK_Down.htm#gene23) | 9 (1.41%) | 0.150618 | 0.700701130 | ko00650 |
| 24 | [Regulation of autophagy](../../../../C:%5CUsers%5Cwujiao%5CDesktop%5C20100324%5Cpathway%5CzsysmvszsyCK_Down.htm#gene24) | 4 (0.63%) | 0.1614949 | 0.719998096 | ko04140 |
| 25 | [Valine, leucine and isoleucine biosynthesis](../../../../C:%5CUsers%5Cwujiao%5CDesktop%5C20100324%5Cpathway%5CzsysmvszsyCK_Down.htm#gene25) | 5 (0.78%) | 0.1798197 | 0.769257775 | ko00290 |
| 26 | [Porphyrin and chlorophyll metabolism](../../../../C:%5CUsers%5Cwujiao%5CDesktop%5C20100324%5Cpathway%5CzsysmvszsyCK_Down.htm#gene26) | 5 (0.78%) | 0.1941836 | 0.769257775 | ko00860 |
| 27 | [Citrate cycle (TCA cycle)](../../../../C:%5CUsers%5Cwujiao%5CDesktop%5C20100324%5Cpathway%5CzsysmvszsyCK_Down.htm#gene27) | 6 (0.94%) | 0.1968385 | 0.769257775 | ko00020 |
| 28 | [Protein export](../../../../C:%5CUsers%5Cwujiao%5CDesktop%5C20100324%5Cpathway%5CzsysmvszsyCK_Down.htm#gene28) | 2 (0.31%) | 0.2013011 | 0.769257775 | ko03060 |
| 29 | [Aminoacyl-tRNA biosynthesis](../../../../C:%5CUsers%5Cwujiao%5CDesktop%5C20100324%5Cpathway%5CzsysmvszsyCK_Down.htm#gene29) | 8 (1.26%) | 0.211242 | 0.779410138 | ko00970 |
| 30 | [Brassinosteroid biosynthesis](../../../../C:%5CUsers%5Cwujiao%5CDesktop%5C20100324%5Cpathway%5CzsysmvszsyCK_Down.htm#gene30) | 3 (0.47%) | 0.2201083 | 0.785052937 | ko00905 |
| 31 | [Galactose metabolism](../../../../C:%5CUsers%5Cwujiao%5CDesktop%5C20100324%5Cpathway%5CzsysmvszsyCK_Down.htm#gene31) | 6 (0.94%) | 0.2368585 | 0.786596131 | ko00052 |
| 32 | [Monoterpenoid biosynthesis](../../../../C:%5CUsers%5Cwujiao%5CDesktop%5C20100324%5Cpathway%5CzsysmvszsyCK_Down.htm#gene32) | 7 (1.1%) | 0.2515863 | 0.786596131 | ko00902 |
| 33 | [Other glycan degradation](../../../../C:%5CUsers%5Cwujiao%5CDesktop%5C20100324%5Cpathway%5CzsysmvszsyCK_Down.htm#gene33) | 4 (0.63%) | 0.2572201 | 0.786596131 | ko00511 |
| 34 | [Sphingolipid metabolism](../../../../C:%5CUsers%5Cwujiao%5CDesktop%5C20100324%5Cpathway%5CzsysmvszsyCK_Down.htm#gene34) | 4 (0.63%) | 0.2572201 | 0.786596131 | ko00600 |
| 35 | [Biotin metabolism](../../../../C:%5CUsers%5Cwujiao%5CDesktop%5C20100324%5Cpathway%5CzsysmvszsyCK_Down.htm#gene35) | 1 (0.16%) | 0.2572978 | 0.786596131 | ko00780 |
| 36 | [Glyoxylate and dicarboxylate metabolism](../../../../C:%5CUsers%5Cwujiao%5CDesktop%5C20100324%5Cpathway%5CzsysmvszsyCK_Down.htm#gene36) | 4 (0.63%) | 0.2757718 | 0.807088565 | ko00630 |
| 37 | [Homologous recombination](../../../../C:%5CUsers%5Cwujiao%5CDesktop%5C20100324%5Cpathway%5CzsysmvszsyCK_Down.htm#gene37) | 6 (0.94%) | 0.2790867 | 0.807088565 | ko03440 |
| 38 | [Cyanoamino acid metabolism](../../../../C:%5CUsers%5Cwujiao%5CDesktop%5C20100324%5Cpathway%5CzsysmvszsyCK_Down.htm#gene38) | 13 (2.04%) | 0.2873314 | 0.809064732 | ko00460 |
| 39 | [Natural killer cell mediated cytotoxicity](../../../../C:%5CUsers%5Cwujiao%5CDesktop%5C20100324%5Cpathway%5CzsysmvszsyCK_Down.htm#gene39) | 4 (0.63%) | 0.3039755 | 0.830209790 | ko04650 |
| 40 | [Lysine biosynthesis](../../../../C:%5CUsers%5Cwujiao%5CDesktop%5C20100324%5Cpathway%5CzsysmvszsyCK_Down.htm#gene40) | 2 (0.31%) | 0.3103588 | 0.830209790 | ko00300 |
| 41 | [Glucosinolate biosynthesis](../../../../C:%5CUsers%5Cwujiao%5CDesktop%5C20100324%5Cpathway%5CzsysmvszsyCK_Down.htm#gene41) | 6 (0.94%) | 0.3228523 | 0.842565759 | ko00966 |
| 42 | [Ubiquitin mediated proteolysis](../../../../C:%5CUsers%5Cwujiao%5CDesktop%5C20100324%5Cpathway%5CzsysmvszsyCK_Down.htm#gene42) | 14 (2.2%) | 0.349429 | 0.877287028 | ko04120 |
| 43 | [Glycerolipid metabolism](../../../../C:%5CUsers%5Cwujiao%5CDesktop%5C20100324%5Cpathway%5CzsysmvszsyCK_Down.htm#gene43) | 6 (0.94%) | 0.3525546 | 0.877287028 | ko00561 |
| 44 | [Biosynthesis of terpenoids and steroids](../../../../C:%5CUsers%5Cwujiao%5CDesktop%5C20100324%5Cpathway%5CzsysmvszsyCK_Down.htm#gene44) | 27 (4.24%) | 0.3871793 | 0.935680998 | ko01062 |
| 45 | [Metabolism of xenobiotics by cytochrome P450](../../../../C:%5CUsers%5Cwujiao%5CDesktop%5C20100324%5Cpathway%5CzsysmvszsyCK_Down.htm#gene45) | 9 (1.41%) | 0.3935107 | 0.935680998 | ko00980 |
| 46 | [Diterpenoid biosynthesis](../../../../C:%5CUsers%5Cwujiao%5CDesktop%5C20100324%5Cpathway%5CzsysmvszsyCK_Down.htm#gene46) | 7 (1.1%) | 0.4193735 | 0.975499228 | ko00904 |
| 47 | [Alanine, aspartate and glutamate metabolism](../../../../C:%5CUsers%5Cwujiao%5CDesktop%5C20100324%5Cpathway%5CzsysmvszsyCK_Down.htm#gene47) | 6 (0.94%) | 0.4347225 | 0.983485655 | ko00250 |
| 48 | [Proteasome](../../../../C:%5CUsers%5Cwujiao%5CDesktop%5C20100324%5Cpathway%5CzsysmvszsyCK_Down.htm#gene48) | 4 (0.63%) | 0.4461335 | 0.983485655 | ko03050 |
| 49 | [Biosynthesis of alkaloids derived from shikimate pathway](../../../../C:%5CUsers%5Cwujiao%5CDesktop%5C20100324%5Cpathway%5CzsysmvszsyCK_Down.htm#gene49) | 16 (2.51%) | 0.4555588 | 0.983485655 | ko01063 |
| 50 | [Benzoxazinoid biosynthesis](../../../../C:%5CUsers%5Cwujiao%5CDesktop%5C20100324%5Cpathway%5CzsysmvszsyCK_Down.htm#gene50) | 6 (0.94%) | 0.4642744 | 0.983485655 | ko00402 |
| 51 | [One carbon pool by folate](../../../../C:%5CUsers%5Cwujiao%5CDesktop%5C20100324%5Cpathway%5CzsysmvszsyCK_Down.htm#gene51) | 2 (0.31%) | 0.4730283 | 0.983485655 | ko00670 |
| 52 | [Oxidative phosphorylation](../../../../C:%5CUsers%5Cwujiao%5CDesktop%5C20100324%5Cpathway%5CzsysmvszsyCK_Down.htm#gene52) | 14 (2.2%) | 0.4808736 | 0.983485655 | ko00190 |
| 53 | [Glutathione metabolism](../../../../C:%5CUsers%5Cwujiao%5CDesktop%5C20100324%5Cpathway%5CzsysmvszsyCK_Down.htm#gene53) | 7 (1.1%) | 0.4871471 | 0.983485655 | ko00480 |
| 54 | [Limonene and pinene degradation](../../../../C:%5CUsers%5Cwujiao%5CDesktop%5C20100324%5Cpathway%5CzsysmvszsyCK_Down.htm#gene54) | 14 (2.2%) | 0.499533 | 0.989815389 | ko00903 |
| 55 | [Biosynthesis of alkaloids derived from ornithine, lysine and nicotinic acid](../../../../C:%5CUsers%5Cwujiao%5CDesktop%5C20100324%5Cpathway%5CzsysmvszsyCK_Down.htm#gene55) | 13 (2.04%) | 0.52362 | 0.999318000 | ko01064 |
| 56 | [DNA replication](../../../../C:%5CUsers%5Cwujiao%5CDesktop%5C20100324%5Cpathway%5CzsysmvszsyCK_Down.htm#gene56) | 5 (0.78%) | 0.5320393 | 0.999318000 | ko03030 |
| 57 | [Glycosylphosphatidylinositol(GPI)-anchor biosynthesis](../../../../C:%5CUsers%5Cwujiao%5CDesktop%5C20100324%5Cpathway%5CzsysmvszsyCK_Down.htm#gene57) | 1 (0.16%) | 0.572694 | 0.999318000 | ko00563 |
| 58 | [Valine, leucine and isoleucine degradation](../../../../C:%5CUsers%5Cwujiao%5CDesktop%5C20100324%5Cpathway%5CzsysmvszsyCK_Down.htm#gene58) | 4 (0.63%) | 0.5781888 | 0.999318000 | ko00280 |
| 59 | [Biosynthesis of alkaloids derived from terpenoid and polyketide](../../../../C:%5CUsers%5Cwujiao%5CDesktop%5C20100324%5Cpathway%5CzsysmvszsyCK_Down.htm#gene59) | 12 (1.88%) | 0.6015937 | 0.999318000 | ko01066 |
| 60 | [Sulfur metabolism](../../../../C:%5CUsers%5Cwujiao%5CDesktop%5C20100324%5Cpathway%5CzsysmvszsyCK_Down.htm#gene60) | 2 (0.31%) | 0.6215714 | 0.999318000 | ko00920 |
| 61 | [Arachidonic acid metabolism](../../../../C:%5CUsers%5Cwujiao%5CDesktop%5C20100324%5Cpathway%5CzsysmvszsyCK_Down.htm#gene61) | 1 (0.16%) | 0.6238966 | 0.999318000 | ko00590 |
| 62 | [Stilbenoid, diarylheptanoid and gingerol biosynthesis](../../../../C:%5CUsers%5Cwujiao%5CDesktop%5C20100324%5Cpathway%5CzsysmvszsyCK_Down.htm#gene62) | 16 (2.51%) | 0.6243524 | 0.999318000 | ko00945 |
| 63 | [Fatty acid biosynthesis](../../../../C:%5CUsers%5Cwujiao%5CDesktop%5C20100324%5Cpathway%5CzsysmvszsyCK_Down.htm#gene63) | 3 (0.47%) | 0.6266521 | 0.999318000 | ko00061 |
| 64 | [Inositol phosphate metabolism](../../../../C:%5CUsers%5Cwujiao%5CDesktop%5C20100324%5Cpathway%5CzsysmvszsyCK_Down.htm#gene64) | 3 (0.47%) | 0.6266521 | 0.999318000 | ko00562 |
| 65 | [Ubiquinone and other terpenoid-quinone biosynthesis](../../../../C:%5CUsers%5Cwujiao%5CDesktop%5C20100324%5Cpathway%5CzsysmvszsyCK_Down.htm#gene65) | 3 (0.47%) | 0.6353734 | 0.999318000 | ko00130 |
| 66 | [alpha-Linolenic acid metabolism](../../../../C:%5CUsers%5Cwujiao%5CDesktop%5C20100324%5Cpathway%5CzsysmvszsyCK_Down.htm#gene66) | 7 (1.1%) | 0.6371748 | 0.999318000 | ko00592 |
| 67 | [Zeatin biosynthesis](../../../../C:%5CUsers%5Cwujiao%5CDesktop%5C20100324%5Cpathway%5CzsysmvszsyCK_Down.htm#gene67) | 3 (0.47%) | 0.6439518 | 0.999318000 | ko00908 |
| 68 | [Mismatch repair](../../../../C:%5CUsers%5Cwujiao%5CDesktop%5C20100324%5Cpathway%5CzsysmvszsyCK_Down.htm#gene68) | 3 (0.47%) | 0.6523867 | 0.999318000 | ko03430 |
| 69 | [Histidine metabolism](../../../../C:%5CUsers%5Cwujiao%5CDesktop%5C20100324%5Cpathway%5CzsysmvszsyCK_Down.htm#gene69) | 2 (0.31%) | 0.6532613 | 0.999318000 | ko00340 |
| 70 | [Phosphatidylinositol signaling system](../../../../C:%5CUsers%5Cwujiao%5CDesktop%5C20100324%5Cpathway%5CzsysmvszsyCK_Down.htm#gene70) | 5 (0.78%) | 0.6600323 | 0.999318000 | ko04070 |
| 71 | [Selenoamino acid metabolism](../../../../C:%5CUsers%5Cwujiao%5CDesktop%5C20100324%5Cpathway%5CzsysmvszsyCK_Down.htm#gene71) | 3 (0.47%) | 0.6688252 | 0.999318000 | ko00450 |
| 72 | [Carotenoid biosynthesis](../../../../C:%5CUsers%5Cwujiao%5CDesktop%5C20100324%5Cpathway%5CzsysmvszsyCK_Down.htm#gene72) | 5 (0.78%) | 0.6790193 | 0.999318000 | ko00906 |
| 73 | [Phenylpropanoid biosynthesis](../../../../C:%5CUsers%5Cwujiao%5CDesktop%5C20100324%5Cpathway%5CzsysmvszsyCK_Down.htm#gene73) | 25 (3.92%) | 0.6872993 | 0.999318000 | ko00940 |
| 74 | [Biosynthesis of plant hormones](../../../../C:%5CUsers%5Cwujiao%5CDesktop%5C20100324%5Cpathway%5CzsysmvszsyCK_Down.htm#gene74) | 34 (5.34%) | 0.6945073 | 0.999318000 | ko01070 |
| 75 | [Peroxisome](../../../../C:%5CUsers%5Cwujiao%5CDesktop%5C20100324%5Cpathway%5CzsysmvszsyCK_Down.htm#gene75) | 8 (1.26%) | 0.7051898 | 0.999318000 | ko04146 |
| 76 | [Non-homologous end-joining](../../../../C:%5CUsers%5Cwujiao%5CDesktop%5C20100324%5Cpathway%5CzsysmvszsyCK_Down.htm#gene76) | 1 (0.16%) | 0.7207948 | 0.999318000 | ko03450 |
| 77 | [Terpenoid backbone biosynthesis](../../../../C:%5CUsers%5Cwujiao%5CDesktop%5C20100324%5Cpathway%5CzsysmvszsyCK_Down.htm#gene77) | 4 (0.63%) | 0.7226751 | 0.999318000 | ko00900 |
| 78 | [Starch and sucrose metabolism](../../../../C:%5CUsers%5Cwujiao%5CDesktop%5C20100324%5Cpathway%5CzsysmvszsyCK_Down.htm#gene78) | 17 (2.67%) | 0.7363995 | 0.999318000 | ko00500 |
| 79 | [Propanoate metabolism](../../../../C:%5CUsers%5Cwujiao%5CDesktop%5C20100324%5Cpathway%5CzsysmvszsyCK_Down.htm#gene79) | 3 (0.47%) | 0.7424505 | 0.999318000 | ko00640 |
| 80 | [Anthocyanin biosynthesis](../../../../C:%5CUsers%5Cwujiao%5CDesktop%5C20100324%5Cpathway%5CzsysmvszsyCK_Down.htm#gene80) | 2 (0.31%) | 0.7515581 | 0.999318000 | ko00942 |
| 81 | [Flavonoid biosynthesis](../../../../C:%5CUsers%5Cwujiao%5CDesktop%5C20100324%5Cpathway%5CzsysmvszsyCK_Down.htm#gene81) | 19 (2.98%) | 0.7958237 | 0.999318000 | ko00941 |
| 82 | [Flavone and flavonol biosynthesis](../../../../C:%5CUsers%5Cwujiao%5CDesktop%5C20100324%5Cpathway%5CzsysmvszsyCK_Down.htm#gene82) | 7 (1.1%) | 0.8070607 | 0.999318000 | ko00944 |
| 83 | [Amino sugar and nucleotide sugar metabolism](../../../../C:%5CUsers%5Cwujiao%5CDesktop%5C20100324%5Cpathway%5CzsysmvszsyCK_Down.htm#gene83) | 5 (0.78%) | 0.8091537 | 0.999318000 | ko00520 |
| 84 | [Biosynthesis of phenylpropanoids](../../../../C:%5CUsers%5Cwujiao%5CDesktop%5C20100324%5Cpathway%5CzsysmvszsyCK_Down.htm#gene84) | 35 (5.49%) | 0.8359223 | 0.999318000 | ko01061 |
| 85 | [Base excision repair](../../../../C:%5CUsers%5Cwujiao%5CDesktop%5C20100324%5Cpathway%5CzsysmvszsyCK_Down.htm#gene85) | 3 (0.47%) | 0.8502516 | 0.999318000 | ko03410 |
| 86 | [Nitrogen metabolism](../../../../C:%5CUsers%5Cwujiao%5CDesktop%5C20100324%5Cpathway%5CzsysmvszsyCK_Down.htm#gene86) | 6 (0.94%) | 0.853474 | 0.999318000 | ko00910 |
| 87 | [Pantothenate and CoA biosynthesis](../../../../C:%5CUsers%5Cwujiao%5CDesktop%5C20100324%5Cpathway%5CzsysmvszsyCK_Down.htm#gene87) | 1 (0.16%) | 0.8587588 | 0.999318000 | ko00770 |
| 88 | [Pentose and glucuronate interconversions](../../../../C:%5CUsers%5Cwujiao%5CDesktop%5C20100324%5Cpathway%5CzsysmvszsyCK_Down.htm#gene88) | 5 (0.78%) | 0.8645796 | 0.999318000 | ko00040 |
| 89 | [Ascorbate and aldarate metabolism](../../../../C:%5CUsers%5Cwujiao%5CDesktop%5C20100324%5Cpathway%5CzsysmvszsyCK_Down.htm#gene89) | 5 (0.78%) | 0.8768494 | 0.999318000 | ko00053 |
| 90 | [Methane metabolism](../../../../C:%5CUsers%5Cwujiao%5CDesktop%5C20100324%5Cpathway%5CzsysmvszsyCK_Down.htm#gene90) | 4 (0.63%) | 0.878726 | 0.999318000 | ko00680 |
| 91 | [Fatty acid metabolism](../../../../C:%5CUsers%5Cwujiao%5CDesktop%5C20100324%5Cpathway%5CzsysmvszsyCK_Down.htm#gene91) | 4 (0.63%) | 0.8878943 | 0.999318000 | ko00071 |
| 92 | [Tyrosine metabolism](../../../../C:%5CUsers%5Cwujiao%5CDesktop%5C20100324%5Cpathway%5CzsysmvszsyCK_Down.htm#gene92) | 3 (0.47%) | 0.9058801 | 0.999318000 | ko00350 |
| 93 | [Nucleotide excision repair](../../../../C:%5CUsers%5Cwujiao%5CDesktop%5C20100324%5Cpathway%5CzsysmvszsyCK_Down.htm#gene93) | 3 (0.47%) | 0.926009 | 0.999318000 | ko03420 |
| 94 | [ABC transporters](../../../../C:%5CUsers%5Cwujiao%5CDesktop%5C20100324%5Cpathway%5CzsysmvszsyCK_Down.htm#gene94) | 5 (0.78%) | 0.9435343 | 0.999318000 | ko02010 |
| 95 | [Tryptophan metabolism](../../../../C:%5CUsers%5Cwujiao%5CDesktop%5C20100324%5Cpathway%5CzsysmvszsyCK_Down.htm#gene95) | 5 (0.78%) | 0.9464346 | 0.999318000 | ko00380 |
| 96 | [Phenylalanine, tyrosine and tryptophan biosynthesis](../../../../C:%5CUsers%5Cwujiao%5CDesktop%5C20100324%5Cpathway%5CzsysmvszsyCK_Down.htm#gene96) | 1 (0.16%) | 0.9470385 | 0.999318000 | ko00400 |
| 97 | [N-Glycan biosynthesis](../../../../C:%5CUsers%5Cwujiao%5CDesktop%5C20100324%5Cpathway%5CzsysmvszsyCK_Down.htm#gene97) | 1 (0.16%) | 0.951372 | 0.999318000 | ko00510 |
| 98 | [Indole alkaloid biosynthesis](../../../../C:%5CUsers%5Cwujiao%5CDesktop%5C20100324%5Cpathway%5CzsysmvszsyCK_Down.htm#gene98) | 1 (0.16%) | 0.9534042 | 0.999318000 | ko00901 |
| 99 | [Steroid biosynthesis](../../../../C:%5CUsers%5Cwujiao%5CDesktop%5C20100324%5Cpathway%5CzsysmvszsyCK_Down.htm#gene99) | 2 (0.31%) | 0.9583674 | 0.999318000 | ko00100 |
| 100 | [Glycerophospholipid metabolism](../../../../C:%5CUsers%5Cwujiao%5CDesktop%5C20100324%5Cpathway%5CzsysmvszsyCK_Down.htm#gene100) | 2 (0.31%) | 0.96391 | 0.999318000 | ko00564 |
| 101 | [RNA degradation](../../../../C:%5CUsers%5Cwujiao%5CDesktop%5C20100324%5Cpathway%5CzsysmvszsyCK_Down.htm#gene101) | 3 (0.47%) | 0.9672207 | 0.999318000 | ko03018 |
| 102 | [Endocytosis](../../../../C:%5CUsers%5Cwujiao%5CDesktop%5C20100324%5Cpathway%5CzsysmvszsyCK_Down.htm#gene102) | 3 (0.47%) | 0.9692657 | 0.999318000 | ko04144 |
| 103 | [Arginine and proline metabolism](../../../../C:%5CUsers%5Cwujiao%5CDesktop%5C20100324%5Cpathway%5CzsysmvszsyCK_Down.htm#gene103) | 1 (0.16%) | 0.986505 | 0.999318000 | ko00330 |
| 104 | [Ribosome](../../../../C:%5CUsers%5Cwujiao%5CDesktop%5C20100324%5Cpathway%5CzsysmvszsyCK_Down.htm#gene104) | 9 (1.41%) | 0.990812 | 0.999318000 | ko03010 |
| 105 | [Cysteine and methionine metabolism](../../../../C:%5CUsers%5Cwujiao%5CDesktop%5C20100324%5Cpathway%5CzsysmvszsyCK_Down.htm#gene105) | 4 (0.63%) | 0.9980626 | 0.999318000 | ko00270 |
| 106 | [Phenylalanine metabolism](../../../../C:%5CUsers%5Cwujiao%5CDesktop%5C20100324%5Cpathway%5CzsysmvszsyCK_Down.htm#gene106) | 1 (0.16%) | 0.9992994 | 0.999318000 | ko00360 |
| 107 | [Spliceosome](../../../../C:%5CUsers%5Cwujiao%5CDesktop%5C20100324%5Cpathway%5CzsysmvszsyCK_Down.htm#gene107) | 10 (1.57%) | 0.999318 | 0.999318000 | ko03040 |
